# Supplementary material for: ADP is the dominant controller of AMP-activated protein kinase activity dynamics in skeletal muscle during exercise
Source: PLoS Comput Biol. 2020 Jul 30;16(7):e1008079. doi: 10.1371/journal.pcbi.1008079 (PMC7433884; doi:10.1371/journal.pcbi.1008079)
Supplement: S1 Text — This file contains Supplementary Methods, Supplementary Tables A-G, Interpretation of S1–S3 Figs, and Supplementary References. (DOCX) [file pcbi.1008079.s001.docx]

**Supplementary information for**

**ADP is the dominant controller of AMP-activated protein kinase activity dynamics in skeletal muscle during exercise**

Ian F. Coccimiglio and David C. Clarke*

Department of Biomedical Physiology and Kinesiology and Centre for Cell Biology, Development and Disease, Simon Fraser University, Burnaby BC V5A 1S6 Canada

* Corresponding author:

Email: [dcclarke@sfu.ca](mailto:dcclarke@sfu.ca)

Tel: 778-782-9777

Fax: 778-782-3040

# Contents:

1. Supplementary Methods
   1. [Model construction: System definition](#Modelconstruct)
   2. [Model construction: Topology](#Modeltopol)
   3. [Model calibration](#Modelcalib)

- 1. [Multi-parametric sensitivity analysis: Criteria for acceptable models](#MPSAcriteria)

- 1. [Multi-parametric sensitivity analysis: Framework for analyzing AMPK activation](#MPSAtheory)

1. [Supplementary Tables A-G](#TableS1)
   1. [Table A. Model species and initial concentrations](#TableS1)
   2. [Table B. Rate equations](#TableS2)
   3. [Table C. System of ordinary differential equations](#TableS3)
   4. [Table D. Model parameter values](#TableS4)
   5. [Table E. Allosteric activation potencies for simulations](#TableS5)
   6. [Table F. Simulation-specific parameter values and initial conditions](#TableS6)
   7. [Table G. Activator properties for the dose-response analysis](#TableS7)
2. [Interpretation of Supplementary Figures S1-S3](#Sup_figs)
3. [Supplementary References](#_Supplementary_References_1)

# Supplementary Methods

## Model construction: System definition

We defined our system as a single muscle fiber contracting within a primary agonist muscle during cycling exercise. Human muscle is composed of three main fiber types that differ according to the predominant myosin heavy-chain isoforms they express (type I, IIa, and IIx). The fiber types differ with respect to their speeds of contraction (slow- or fast-twitch), their metabolism (oxidative or glycolytic), and their fatiguability (fatigue-resistant, fatigable) [1]. The recruitment of the fibers depends on the task, such that we had to consider the fiber type in our study. We assumed that our muscle fiber features average contractile, metabolic, and fatigability properties in the spectrum from slow to fast-twitch fibers, and thus would most closely resemble a type-IIa fiber [2]. This assumption had two benefits. First, it allowed us to specify relatively simple recruitment profiles in response to distinct exercise modalities. In our study, we simulated two distinct types of exercise, namely continuous submaximal-intensity exercise and sprint-interval exercise (SIE), the latter of which involves repeated maximum-intensity 30-s sprints interspersed with 4-min-long passive recovery bouts. Type IIa fibers are recruited in both types of exercise [3–5], and we thus assumed that the fiber was recruited throughout the duration of exercise in a manner corresponding to the measured power profiles. Second, the assumption also enabled the valid comparison of model outputs to data from homogenates of human muscle biopsies. Biopsies contain a mixture of muscle fiber types and therefore the data represent the average response of the fibers within the biopsy. Additional simplifying assumptions included that the muscle fiber behaved as a well-stirred tank reactor and the modeled molecules were assumed to be present in sufficient concentrations so as to behave deterministically. These assumptions enabled the use of ordinary differential equations (ODEs) as the model’s mathematical framework.

## Model construction: Topology

Our model features three modules, two of which represent biochemical reactions intrinsic to the cell (*the bioenergetic module* and the *AMPK regulatory module*), as well as a *pharmacological-activator (PA) module*, which represents reactions involving exogenously added small-molecule activators of AMPK (Fig. 1 in the main text). The bioenergetic, AMPK regulatory, and PA modules respectively feature five, eight, and four molecular species (Table A). These species are interconverted through intermolecular association and enzyme-catalyzed biochemical reactions, the rate equations for which are listed in Table B. These rate equations were used within the 17 coupled nonlinear ODEs that describe the rates of change of the concentrations of the molecular species (Table C). The species concentrations are given by sum of the reactions that generate and consume each species, expressed mathematically as follows:

| $\frac{d}{dt}[species]=\sum{rates}_{generation}-\sum{rates}_{consumption}$ | (A) |
| --- | --- |

The rate equations associated with the bioenergetic and AMPK-regulatory modules feature 47 kinetic parameters while the PA module features ten additional parameters (Table D). The initial concentrations for each species in the model are listed in Table A. We converted concentrations reported in units of mmol/kg dry weight of muscle to mmol/L by multiplying by 3.13 L/kg [6].

*The bioenergetic module*. The bioenergetic module is based on the previously validated model of Vicini et al. [7], and incorporates the adenylate kinase (AK) reaction from the model of Lambeth et al. [8]. This module explicitly models ATP hydrolysis (Fig. 1 and Table B, reaction r1) and the ATP supply processes of oxidative phosphorylation (Fig. 1 and Table B, reaction r2), the creatine kinase reaction (CK; Fig. 1 and Table B, reactions r3 and r4), and the AK reaction (Fig. 1 and Table B, reaction r5). The rate equations and pre-calibration initial conditions for these reactions were obtained from the source models [7,8].

The bioenergetic module features the following simplifications to foster parsimony. First, we assumed a constant total adenine nucleotide pool: we omitted the AMP degradation reaction to inosine monophosphate (IMP) and ammonia [9] and the various counterbalancing synthesis reactions. Second, we did not include glycolysis and lactate formation, which supply ATP and NADH. We also omitted modeling dynamic changes in pH but instead set pH at a constant level. These simplifications were justified on theoretical grounds because the primary purpose of the bioenergetic module was to satisfactorily replicate AXP dynamics, which served as inputs into the AMPK regulatory module, rather than faithfully replicate all aspects of muscle metabolism. We empirically validated these assumptions by comparing the model predictions to data and by performing sensitivity analyses, as described in the Results.

Exercise was simulated by increasing the rate of ATP hydrolysis. Specifically, we changed the value of the rate constant in reaction r1 (Table B) from a value reflecting the rate at rest (*k_rest_*, parameter 12 in Table D) to one commensurate with exercise (“stimulated”, *k_stim_*, parameter 13 in Table D). Simulated exercise was ceased by setting the rate constant of the ATP hydrolysis reaction to *k_post_* (parameter 14 in Table D) [7]. We simulated exercise protocols of different intensities by adjusting *k_stim_* until the simulated PCr concentrations matched the values measured in muscle biopsies, as PCr concentration is a well-established marker of muscle fiber recruitment and exercise intensity [10–12].

*The AMPK regulatory module*. The AMPK regulatory module featured the putative mechanisms of AXP-mediated control of AMPK activity (Fig. 1). The AMPK regulatory module consisted of eight species: unmodified and phosphorylated AMPK that were either unbound or bound to one of AMP, ADP, or ATP (Fig. 1). Our naming convention is as follows: complexes are written as AXP-(p)-AMPK, wherein the AXP is one of AMP, ADP, or ATP, the “p” indicates phosphorylation, and the suffix denotes the AMPK protein. For example, *AMP-bound phospho-AMPK* is written as *AMP-p-AMPK*, whereas *ADP bound to unphosphorylated AMPK* is written as *ADP-AMPK*.

We made the following simplifying assumptions to foster model parsimony: 1) cells contain only a single isoform of AMPK, 2) AMPK reversibly binds to the AXP at a single binding site (Table B, reactions r6 to r11), 3) AMPK features a single activating phosphorylation site (corresponding to the Thr-172 residue), and 4) the kinetics of AMPK phosphorylation and dephosphorylation could be adequately described using Michaelis-Menten kinetics (Table B, reactions r12 to r19).

These assumptions were justified as follows. First, while isoform-specific responses to exercise exist [13], each isoform is activated during exercise [14], such that we considered aggregate AMPK activity as the key factor driving training adaptations. Second, AXP-(p)-AMPK binding kinetics are well represented by a first-order process indicating a single binding site [15,16], which is thought to be a high-affinity AXP-binding site at the CBS3 (cystathionine-β-synthase) motif on AMPK [17]. Third, the primary mechanism of AMPK activation in mammalian cells is its phosphorylation at the Thr-172 residue [18,19]. The assumption of Michaelis-Menten kinetics was justified as follows. AMPK phosphorylation and dephosphorylation reactions each involve an enzyme and two reactants: AMPK phosphorylation involves the kinase (LKB1 or CaMKKβ), [(AXP)-AMPK], and ATP while phospho-AMPK dephosphorylation involves a phosphatase (PP1, PP2A, or PP2C [14]), (AXP)-p-AMPK, and H_2_O. We assume that ATP and H_2_O concentrations are relatively constant and well in excess of the other reactants. We assumed that the kinase and phosphatase concentrations remain constant due to the transition complexes existing only for very short times, and that they are constitutively active, as has been demonstrated for LKB1 [20]. The effects of AXP binding on the AMPK phosphorylation and dephosphorylation reaction rates were modeled by using different *V_max_* values for each of the AXP-(p)-AMPK species (Table D, parameters 40-47).

We quantified AMPK complex activities as *V_max_* values, which were calculated as the product of complex-specific *k_cat_* and concentration values, the latter of which varied dynamically over time. We assumed that only phosphorylated AMPK complexes were active [18,19]. In addition, AMPK is allosterically activated by AMP, which we modeled by increasing the *k_cat_* for AMP-p-AMPK complex relative to the *k_cat_* values of the other complexes (Table E).

With respect to initial conditions, we set the total AMPK concentration equal to 0.6 mM. This choice was justified by the observations that the AMPK subunit β1 concentration is 0.06 mM in rat extensor digitorum longus (EDL) muscle, and is present in ~10% of the AMPK molecules, with the balance featuring the β2 isoform [21,22]. This total AMPK concentration was then assumed to be composed of equal concentrations of the six AXP-(p)-AMPK complexes, which resulted in their pre-calibration initial conditions (Table A).

*The pharmacological-activator module*. The PA module adds the influence of pharmacological agents that directly bind to AMPK. Here, we modeled the activation of AMPK by the small-molecule activators 5-aminoimidazole-4-carboxamide ribonucleoside (AICAR) and Compound 991 (C991). We set the topology of the module to parsimoniously represent the experiments from published studies of AMPK activation in response to AICAR and C991 treatment. In the case of AICAR, the experiment involved perfusion of rat gastrocnemius muscle with AICAR, which is taken up by the cells and phosphorylated into the AMP analogue AICAR 5’-monophosphate (ZMP) by the enzyme adenosine kinase [23]. We introduced reactions representing the interconversion of AICAR to ZMP and the degradation of ZMP (Fig. 1 and Table B, reactions r24 and r25). We then added the reversible binding reaction of ZMP with AMPK and p-AMPK (Fig. 1 and Table B, r22 and r23).

The experiment with C991 involved adding bolus doses of C991 (10^-2^ mM, 10^-3^ mM, or 10^-4^ mM) to the culture media overlying C2C12 murine myotube cells [24]. The action of C991 on AMPK was modeled as a binding reaction (Fig. 1 and Table B, reactions r22 and r23). We made the simplifying assumption that both ZMP and C991 bind competitively to the AXP-binding site on AMPK, which is only true for ZMP because C991 actually binds to the allosteric drug and metabolite-binding (ADaM) site located at the interface of the kinase- and glycogen-binding domains of the α- and β-subunits, respectively [25]. We considered only β2-containing AMPK isoforms as the active complexes.

## Model calibration

The model parameter values were calibrated using the procedure outlined by Kim et al. [26]. We first located published parameter estimates, with those for the bioenergetics module obtained from the source models [7,8] (Table D, parameters 1-19). For the AMPK regulatory module (Table D, parameters 20-31), the kinetic parameter values for AMP and ADP binding to AMPK were set by assuming that the forward binding constants (*k_f_*) equaled 1 mM^-1^s^-1^ and then calculating the reverse binding constants (*k_r_*) from the product of this *k_f_* value and the K_D_ values reported by Xiao et al. [16] (Table D). Those for ATP were set to reflect the K_D_ for Mg-ATP^2-^ binding to AMPK [27], given that most ATP is coordinated with magnesium *in vivo*. This K_D_ is substantially less than that of unconjugated ATP [16]. The same values were used for AXP binding to phospho-AMPK (Table D) [16,28]. In some cases the published values could be straightforwardly used, in other cases the units required conversion using biochemical equations (Table D) [29]. These calculations are provided in the Supplementary Spreadsheet file. These initial estimates were collectively called the “pre-calibration” parameter values.

Most of the pre-calibration parameter values were derived from *in vitro* experiments, such that the *in vivo* values might be different. We therefore sought to adjust the parameter values so that the model reproduced measurements made from exercising humans. We used the data of Stephens et al., which features time course measurements of PCr and AXP concentrations and AMPK activity in muscle biopsies of human volunteers who cycled for 30 min at moderate intensity (~63% of $\dot{V}O_{2peak}$) [30]. We also mandated that the model replicate the qualitative patterns of the phospho-AMPK time course observed during submaximal aerobic exercise. These features included the following: 1) at least 25% of total AMPK was phosphorylated at rest [31], 2) in response to the onset of moderate-intensity exercise, phospho-AMPK levels increased gradually and substantially, and 3) upon cessation of exercise, phospho-AMPK levels returned to resting levels [32,33]. To determine the parameters that most affect the phospho-AMPK kinetics, we performed a one-factor-at-a-time local sensitivity analysis. The analysis involved adjusting each parameter value and initial condition by 10% from the pre-calibration value, simulating the model, and documenting the observed change on the features of the phospho-AMPK time course (data not shown). The parameters that had the highest effects on the features were then adjusted until the three criteria were satisfied.

The model was then manually adjusted to fit the activity data [30]. Kinase activities are typically expressed in absolute units specific to a peptide substrate (i.e., mol of substrate phosphorylated per unit volume per unit time), or as fold changes relative to another condition. The absolute units are specific to the *in vitro* situation and therefore do not necessarily translate directly to the *in vivo* situation. Therefore, we set the first data point equal to the V_max_ computed from the model initial conditions (in units of mmol L^-1^ s^-1^), followed by converting the subsequent data points into absolute activities by computing the products of the initial *V_max_* and the reported fold-changes in activity. In this way, the numbers predicted for the AMPK activities are not necessarily “real” in an absolute sense, but they do faithfully reflect the experimentally observed changes. The resulting parameter set is referred to as the “calibrated parameter set” (Table D).

The kinetic parameter values for reactions involving AMPK and ZMP or C991 are listed in Tables D, E, and F, and were obtained from the literature or expressed as a value relative to the corresponding parameters for AMP. Simulating the effects of ZMP introduced the complication of its precursor AICAR being the experimental treatment, such that we needed to include the conversion reaction in the model. Furthermore, the dataset used for comparison was from experiments involving AICAR perfusion of rat skeletal muscle, which necessitated an uptake reaction. We modeled these reactions using unidirectional first-order rate equations and manually tuned the parameter values so that the predicted ZMP concentration kinetics matched available ZMP time-course concentration data [34]. No calibration was performed for the parameters affecting AMPK activity when simulating the activator time courses. The pharmacological module was also used for simulating the dose-responses for each of the activators (AMP, ADP, ZMP, C991), and the corresponding activator K_D_s, potencies, and concentration ranges employed in this analysis are listed in Table G [28].

## *Multi-parametric sensitivity analysis: Criteria for acceptable models*

Randomly generated parameter sets can lead to numerical pathologies or biologically implausible model outputs, so we specified the following qualitative criteria to classify models as acceptable:

- All concentrations were positive values throughout the duration of the simulation.
- All model outputs were real numbers.
- Input parameters did not result in matrices that were singular or badly scaled.
- The time course ran to completion (t > 2,780 s out of a 2,800 s simulation).
- The initial amount of phospho-AMPK was less than 40% of the total AMPK.
- The initial amount of phospho-AMPK was greater than 1% of the total AMPK.
- The maximum amount of phospho-AMPK was at least 20% greater than the initial amount of phosphorylated AMPK.
- The maximum amount of phospho-AMPK during stimulation was greater than 1% of the total amount of AMPK.
- The final amount of phospho-AMPK was greater than the resting amount of AMPK.
- Phospho-AMPK levels were at steady state prior to the start of exercise [changed by less than 0.001 mM from 30 s before stimulation]
- Total AMPK concentration was conserved (did not change more than 2 µM throughout the simulation).

## *Multi-parametric sensitivity analysis: F*ramework for analyzing AMPK activation

We expect that interactions between parameters determine the dominance of a given AXP in controlling AMPK activity, but the number of parameters precludes straightforward analysis. We therefore proposed a framework for the sensitivity analysis based on simplified theoretical considerations. We first considered a reaction system containing phospho-AMPK and an allosteric activator of AMPK. At steady state, the concentration of activator-bound phospho-AMPK can be expressed as follows:

| $\left[ act\cdot p\text{-}AMPK \right]=\frac{\left[ act \right]\left[ \text{p-AMPK} \right]}{K_{D,actpAMPK}}$ | (B) |
| --- | --- |

where $\left[ act\cdot p\text{-}AMPK \right]$ is the concentration of activator-bound phospho-AMPK, $K_{D,actpAMPK}$ is the dissociation constant for activator-bound phospho-AMPK, $\left[ act \right]$ is the concentration of activator, and $\left[ p\text{-}AMPK \right]$ is the concentration of phospho-AMPK. Note that the concentrations are those observed after steady state has established, not the concentrations that were added to the system at the start of the reaction.

We next defined $\alpha_{\Sigma}$, the total AMPK activity of the system if a substrate were present in saturating amounts, as the following sum:

| $\alpha_{\Sigma}=V_{actpAMPK}\left[ act\cdot p\text{-}AMPK \right]+V_{pAMPK}\left[ p\text{-}AMPK \right]$ | (C) |
| --- | --- |

in which $V_{actpAMPK}$ and $V_{pAMPK}$ are the V_max_ of $act\cdot p\text{-}AMPK$ and $p\text{-}AMPK$ respectively. Substituting Equation B into Equation C gives

| $\alpha_{\Sigma}=V_{actpAMPK}\frac{\left[ act \right]\left[ \text{p-AMPK} \right]}{K_{D,actpAMPK}}+V_{pAMPK}\left[ p\text{-}AMPK \right]$ | (D) |
| --- | --- |

which upon simplification gives

| $\alpha_{\Sigma}=\left[ \text{p-AMPK} \right]\left( V_{actpAMPK}\frac{\left[ act \right]}{K_{D,actpAMPK}}+V_{pAMPK} \right)$ | (E) |
| --- | --- |

Phosphorylation and allostery activate AMPK in a multiplicative manner [35], such that $V_{actpAMPK}$ can be expressed as a product of allosteric activation, $\alpha_{allo}$, and $V_{pAMPK}$. Substituting this product into Equation E gives

| $\alpha_{\Sigma}=\left[ \text{p-AMPK} \right]\left( V_{pAMPK}\alpha_{allo}\frac{\left[ act \right]}{K_{D,actpAMPK}}+V_{pAMPK} \right)$ | (F) |
| --- | --- |

such that

| $\alpha_{\Sigma}=V_{pAMPK}\left[ \text{p-AMPK} \right]\left( \alpha_{allo}\frac{\left[ act \right]}{K_{D,actpAMPK}}+1 \right)$ | (G) |
| --- | --- |

Equation G expresses the five factors that determine an activator’s potency for activating AMPK, i.e., the concentration of activator, the binding affinity of the activator for AMPK (expressed as K_D_), the concentration of phospho-AMPK and its activity, and the allosteric activation of AMPK provided by the activator.

One way to compare the relative potencies of two activators (denoted by the subscripts 1 and 2) is to express as a ratio the equations for the two activators:

| $\frac{\alpha_{\Sigma,1}}{\alpha_{\Sigma,2}}=\frac{V_{pAMPK}\left[ \text{p-AMPK} \right]_{1}\left( \alpha_{allo,1}\frac{\left[ {act}_{1} \right]}{K_{D1}}+1 \right)}{V_{pAMPK}\left[ \text{p-AMPK} \right]_{2}\left( \alpha_{allo,2}\frac{\left[ {act}_{2} \right]}{K_{D2}}+1 \right)}$ | (H) |
| --- | --- |

Since $V_{pAMPK}$ is the same regardless of the activator, the final form is

| $\frac{\alpha_{\Sigma,1}}{\alpha_{\Sigma,2}}=\frac{\left[ \text{p-AMPK} \right]_{1}\left( \alpha_{allo,1}\frac{\left[ {act}_{1} \right]}{K_{D1}}+1 \right)}{\left[ \text{p-AMPK} \right]_{2}\left( \alpha_{allo,2}\frac{\left[ {act}_{2} \right]}{K_{D2}}+1 \right)}$ | (I) |
| --- | --- |

The benefits of Equation I are that it compactly expresses four factors that are each determined by individual parameters or intuitive groupings from the AMPK model. Specifically, $\alpha_{allo}$ is specified as an independent parameter in the model (Table E), the *k_f_* and *k_r_* forward and reverse rate constants for AXP binding to AMPK and phospho-AMPK determine the K_D_, while $\left[ act \right]$ is determined primarily by the bioenergetic reactions. Furthermore, computing compound ratios of these parameters or groupings of parameters that pertain to different activators should provide insights into their sensitivities.

While useful for organizing our sensitivity analysis, the above equations *cannot* be directly used to validly estimate AMPK activities. In a real system containing AMPK kinases and phosphatases, the determination of $\left[ \text{p-AMPK} \right]$ is complex because its levels are determined by the activators and their propensities to enhance upstream kinase activity and reduce phosphatase activity towards AMPK. Accordingly, $\left[ \text{p-AMPK} \right]$ depends on the enzyme kinetic parameters of AMPK kinases and phosphatases, as well as on $\left[ act \right]$ and the K_D_. In addition, the presence of ATP in the system causes further complexities because it is both necessary for kinase activity (as a substrate) but also competes with AMP and ADP for binding. The complexities of the real system make it difficult to express AMPK activity control as a tractable analytical equation and emphasizes the need for kinetic models to study it.

# Supplementary Tables

**Table A**. Model species and initial concentrations. Model species (state variables) and their initial concentrations before and after the model calibration.

| **Species** | **Name** | **Pre-calibration Value (mM)** | **Calibrated Value (mM)** | **Basis for values** | | | |
| --- | --- | --- | --- | --- | --- | --- | --- |
|  |  |  |  | Ref | Calc | Calib | Est |
| *Bioenergetic module* | | | | | | | |
| $x(1)$ | ATP | 8.2 [7] | 7.66 | ☒ | ☐ | ☒ | ☐ |
| $x(2)$ | ADP | 1.3×10^-2^ [7] | 5.68×10^-2^ | ☒ | ☐ | ☒ | ☐ |
| $x(3)$ | AMP | 2.0×10^-5^ [8] | 4.44×10^-4^ | ☒ | ☐ | ☒ | ☐ |
| $x(4)$ | PCr | 32.1 [7] | 22.1 | ☒ | ☐ | ☒ | ☐ |
| $x(5)$ | P_i_ | 3.183 [7] | 3.183 | ☒ | ☐ | ☐ | ☐ |
| *AMPK regulatory module* | | | | | | | |
| $x(6)$ | ATP-AMPK* | 1×10^-1^ [21] | 0.075 | ☒ | ☒ | ☒ | ☐ |
| $x(7)$ | ADP-AMPK* | 1×10^-1^ [21] | 0.075 | ☒ | ☒ | ☒ | ☐ |
| $x(8)$ | AMP-AMPK* | 1×10^-1^ [21] | 0.075 | ☒ | ☒ | ☒ | ☐ |
| $x(9)$ | ATP-p-AMPK* | 1×10^-1^ [21] | 0.075 | ☒ | ☒ | ☒ | ☐ |
| $x(10)$ | ADP-p-AMPK* | 1×10^-1^ [21] | 0.075 | ☒ | ☒ | ☒ | ☐ |
| $x(11)$ | AMP-p-AMPK* | 1×10^-1^ [21] | 0.075 | ☒ | ☒ | ☒ | ☐ |
| $x(12)$ | AMPK* | 0 | 0.075 | ☒ | ☒ | ☒ | ☐ |
| $x(13)$ | p-AMPK* | 0 | 0.075 | ☒ | ☒ | ☒ | ☐ |
| *Pharmacological activator module*** | | | | | | | |
| $x$(14) | Act-AMPK | 0 | | ☐ | ☐ | ☐ | ☒ |
| $x$(15) | Act-p-AMPK | 0 | | ☐ | ☐ | ☐ | ☒ |
| $x$(16) | Act | 0 | | ☐ | ☐ | ☐ | ☒ |
| $x$(17) | AICAR | 0 | | ☐ | ☐ | ☐ | ☒ |

Ref, references; Calc, calculations; Calib, calibrations; Est, estimations.

* Total AMPK was set to 0.6 mM and divided equally between the six nucleotide-bound complexes prior to calibration. We found that the initial concentration had no effect on outcomes, and so we divided all AMPK complexes equally post-calibration.

**The initial parameter values were based on ZMP, values for Compound 991 are presented in Table F.

**Table B.** Rate equations.

| **Description** | **Equation** |
| --- | --- |
| Rate of ATP hydrolysis | $r1=k_{stim}\times x\left( 1 \right)$  or  $r1=k_{rest}\times x\left( 1 \right)$ |
| Oxidative Phosphorylation | $r2=\frac{V_{maxOxPhos}\times\left( \frac{x\left( 2 \right)}{K_{ADP}} \right)^{nH}}{1+\left( \frac{x(2)}{K_{ADP}} \right)^{nH}}$ |
| Forward Creatine Kinase | $r3=\frac{\frac{V_{forCK}\times x\left( 2 \right)\times x\left( 4 \right)}{K_{ia}\times K_{b}}}{1+\frac{x\left( 2 \right)}{K_{ia}}+\frac{x\left( 4 \right)}{K_{ib}}+\frac{x\left( 1 \right)}{K_{iq}}+\frac{x\left( 2 \right)\times x\left( 4 \right)}{K_{ia}\times K_{b}}+\frac{\left( TCr-x\left( 4 \right) \right)\times x\left( 1 \right)}{K_{iq}\times K_{p}}}$ |
| Reverse Creatine Kinase | $V_{revCK}= \frac{V_{forCK}\times K_{iq}\times K_{p}}{K_{eqCK}\times K_{ia}\times K_{b}}$  $r4= \frac{\frac{V_{revCK}\times x\left( 1 \right)\times\left( TCr-x\left( 4 \right) \right)}{K_{iq}\times K_{p}}}{1+\frac{x\left( 2 \right)}{K_{ia}}+\frac{x\left( 4 \right)}{K_{ib}}+\frac{x\left( 1 \right)}{K_{iq}}+\frac{x\left( 2 \right)\times x\left( 4 \right)}{K_{ia}\times K_{b}}+\frac{\left( \left( TCr-x\left( 4 \right) \right)\times x\left( 1 \right) \right)}{K_{iq}\times K_{p}}}$ |
| Forward Adenylate Kinase | $r5f=\frac{\frac{V_{forAK}\times x(1)}{kmt*kmm}}{1+\frac{x\left( 1 \right)}{kmt}+\frac{x\left( 3 \right)}{kmm}+\frac{x\left( 1 \right)\times x\left( 3 \right)}{kmt\times kmm}+\frac{2*x\left( 2 \right)}{kmd}+\frac{\left( x\left( 2 \right) \right)^{2}}{{kmd}^{2}}}$ |
| Reverse Adenylate Kinase | $V_{revAK}=\frac{V_{forAK}\times{kmd}^{2}}{K_{eqAK}\times kmt\times kmm}$  $r5r=\frac{\frac{V_{revAK}\times\left( x\left( 2 \right) \right)^{2}}{{kmd}^{2}}}{1+\frac{x\left( 1 \right)}{kmt}+\frac{x\left( 3 \right)}{kmm}+\frac{x\left( 1 \right)\times x\left( 3 \right)}{kmt\times kmm}+\frac{2*x\left( 2 \right)}{kmd}+\frac{\left( x\left( 2 \right) \right)^{2}}{{kmd}^{2}}}$ |
| AMPK binding ATP | $r6=k6f\times x\left( 1 \right)\times x\left( 12 \right)-k6r\times x(6)$ |
| AMPK binding ADP | $r7= k7f\times x\left( 2 \right)\times x\left( 12 \right)-k7r\times x(7)$ |
| AMPK binding AMP | $r8=k8f\times x\left( 3 \right)\times x\left( 12 \right)-k8r\times x(8)$ |
| p-AMPK binding ATP | $r9=k9f\times x\left( 1 \right)\times x\left( 13 \right)-k9r\times x(9)$ |
| p-AMPK binding ADP | $r10=k10f\times x\left( 2 \right)\times x\left( 13 \right)-k10r\times x(10)$ |
| p-AMPK binding AMP | $r11=k11f\times x\left( 3 \right)\times x\left( 13 \right)-k11r\times x\left( 11 \right)$ |
| Phosphorylation  AMPK | $r12=\frac{V_{maxKinase}\times x\left( 12 \right)}{km12+x(12)}$ |
| Dephosphorylation  p-AMPK | $r13=\frac{V_{maxPPase}\times x\left( 13 \right)}{km13+x\left( 13 \right)}$ |
| Phosphorylation  ATP-AMPK | $r14= \frac{V_{maxKinaseATP}\times x\left( 6 \right)}{km14+x(6)}$ |
| Dephosphorylation  ATP-p-AMPK | $r15=\frac{V_{maxPPaseATP}\times x\left( 9 \right)}{km15+x\left( 9 \right)}$ |
| Phosphorylation  ADP-AMPK | $r16=\frac{V_{maxKinaseADP}\times x\left( 7 \right)}{km16+x(7)}$ |
| Dephosphorylation  ADP-p-AMPK | $r17=\frac{V_{maxPPaseADP}\times x\left( 10 \right)}{km17+x\left( 10 \right)}$ |
| Phosphorylation  AMP-AMPK | $r18=\frac{V_{maxKinaseAMP}\times x\left( 8 \right)}{km18+x(8)}$ |
| Dephosphorylation  AMP-p-AMPK | $r19=\frac{V_{maxPPaseAMP}\times x\left( 11 \right)}{km19+x\left( 11 \right)}$ |
| Phosphorylation  Activator-AMPK | $r20=\frac{V_{maxKinaseZMP}\times x(14)}{km20+x(14)}$ |
| Dephosphorylation  Activator-p-AMPK | $r21= \frac{V_{maxPPaseZMP}\times x(15)}{km21 + x(15)}$ |
| AMPK binding Activator | $r22= k12f\times x\left( 12 \right)\times x\left( 16 \right)-k12r\times x\left( 14 \right)$ |
| p-AMPK binding Activator | $r23=k13f\times x\left( 13 \right)\times x\left( 16 \right)-k13r\times x\left( 15 \right)$ |
| AICAR forming Activator | $r24=k_{AICAR}\times x(17)$ |
| Activator degradation rate | $r25=k_{Act}\times x\left( 16 \right)$ |

**Table C.** System of ordinary differential equations.

| **Variable** | **Differential equation (with respect to time)** |
| --- | --- |
| ATP | dx(1) = − r1 + r2 − CK − AK − r6 − r9; |
| ADP | dx(2) = + r1 − r2 + CK + 2×AK − r7 − r10; |
| AMP | dx(3) = − AK − r8 − r11 − r20; |
| PCr | dx(4) = + CK; |
| Pi | dx(5) = + r1 − r2; |
| ATP-AMPK | dx(6) = + r6 − r14 + r15; |
| ADP-AMPK | dx(7) = + r7 − r16 + r17; |
| AMP-AMPK | dx(8) = + r8 − r18 + r19; |
| ATP-p-AMPK | dx(9) = + r9 + r14 − r15; |
| ADP-p-AMPK | dx(10) = + r10 + r16 − r17; |
| AMP-p-AMPK | dx(11) = + r11 + r18 − r19; |
| AMPK | dx(12) = − r6 − r7 − r8 − r12 + r13; |
| p-AMPK | dx(13) = − r9 − r10 − r11 + r12 - r13; |
| Activator-AMPK | dx(14) = − r20 − r21 + r22; |
| Activator-p-AMPK | dx(15) = + r20 - r21 + r23; |
| Activator | dx(16) = − r22 − r23 + r24 − r25;* |
| AICAR | dx(17) = 0; |

*Activator uptake and depletion rates were set to zero when simulating dose-response experiments.

**Table D**. Model parameter values.

| **Reaction** | **(#) Kinetic parameter** | **Pre-calibration value** | **Calibrated value** | **Units** | **Basis for values** | | | |
| --- | --- | --- | --- | --- | --- | --- | --- | --- |
|  |  |  |  |  | Ref* | Calc | Calib | Est |
| *Bioenergetic module* | | | | | | | | |
| Creatine kinase | (1) V_forCK_ | 1.00×10^2^ [7] | 1.00×10^2^ | mM/s | ☒ | ☐ | ☐ | ☐ |
|  | (2) K_b_ | 1.11 [7] | 1.11 | mM | ☒ | ☐ | ☐ | ☐ |
|  | (3) K_ia_ | 0.135 [7] | 0.135 | mM | ☒ | ☐ | ☐ | ☐ |
|  | (4) K_ib_ | 3.9 [7] | 3.9 | mM | ☒ | ☐ | ☐ | ☐ |
|  | (5) K_iq_ | 3.5 [7] | 3.5 | mM | ☒ | ☐ | ☐ | ☐ |
|  | (6) K_p_ | 3.8 [7] | 3.8 | mM | ☒ | ☐ | ☐ | ☐ |
|  | (7) K_eqCK_ | 1.77×10_­­_^(9-pH)^ [7] | 1.77×10_­­_^(9-pH)^ | unitless | ☒ | ☐ | ☐ | ☐ |
|  | (8) TCr | 42 [7] | 39 | mM | ☒ | ☐ | ☐ | ☐ |
| Oxidative phosphorylation | (9) K_ADP_ | 5.8×10^-2^ [7] | 5.8×10^-2^ | mM | ☒ | ☐ | ☐ | ☐ |
|  | (10) V_maxOxPhos_ | 0.5 [7] | 0.5 | mM/s | ☒ | ☐ | ☐ | ☐ |
|  | (11) nH | 2.568 [7] | 2.568 | unitless | ☒ | ☐ | ☐ | ☐ |
| ATP hydrolysis | (12) k_rest_ | 1.4×10^-3^ [7] | 2.6×10^-2^ | 1/s | ☒ | ☐ | ☒ | ☐ |
|  | (13) k_stim_ | 1.39×10^-2^ [7] | 5.0×10^-2^ | 1/s | ☒ | ☐ | ☒ | ☐ |
|  | (14) k_post_ | NA | 2.6×10^-2^ | 1/s | ☐ | ☐ | ☐ | ☒ |
| Adenylate kinase | (15) V_forAK_ | 14.66 [8] | 14.66 | mM/s | ☒ | ☒ | ☐ | ☐ |
|  | (16) kmt | 0.27 [8] | 0.27 | mM | ☒ | ☐ | ☐ | ☐ |
|  | (17) kmd | 0.35 [8] | 0.35 | mM | ☒ | ☐ | ☐ | ☐ |
|  | (18) kmm | 0.32 [8] | 0.32 | mM | ☒ | ☐ | ☐ | ☐ |
|  | (19) K_eqADK_ | 2.21 [8,36] | 0.744 | unitless | ☒ | ☐ | ☒ | ☐ |
| *AMPK regulatory module* | | | | | | | | |
| ATP-AMPK | (20) k6f | 1 [16] | 1 | 1/(mM×s) | ☒ | ☒ | ☒ | ☐ |
|  | (21) k6r | 1.8×10^-2^ [16] | 2.2×10^-1^ | 1/s | ☒ | ☒ | ☒ | ☐ |
| ADP-AMPK | (22) k7f | 1 [16] | 1.5 | 1/(mM×s) | ☒ | ☒ | ☒ | ☐ |
|  | (23) k7r | 1.5×10^-3^ [16] | 2.25×10^-3^ | 1/s | ☒ | ☒ | ☒ | ☐ |
| AMP-AMPK | (24) k8f | 1 [16] | 1.5 | 1/(mM×s) | ☒ | ☒ | ☒ | ☐ |
|  | (25) k8r | 2.5×10^-3^ [16] | 3.75×10^-3^ | 1/s | ☒ | ☒ | ☒ | ☐ |
| ATP-p-AMPK | (26) k9f | 1 [16] | 1 | 1/(mM×s) | ☒ | ☒ | ☒ | ☐ |
|  | (27) k9r | 1.8×10^-2^ [16] | 4.0×10^-1^ | 1/s | ☒ | ☒ | ☒ | ☐ |
| ADP-p-AMPK | (28) k10f | 1 [16] | 1.5 | 1/(mM×s) | ☒ | ☒ | ☒ | ☐ |
|  | (29) k10r | 1.5×10^-3^ [16] | 2.25×10^-3^ | 1/s | ☒ | ☒ | ☒ | ☐ |
| AMP-p-AMPK | (30) k11f | 1 [16] | 1 | 1/(mM×s) | ☒ | ☒ | ☒ | ☐ |
|  | (31) k11r | 2.5×10^-3^ [16] | 3.75×10^-3^ | 1/s | ☒ | ☒ | ☒ | ☐ |
| ATP-(p)-AMPK kinase and phosphatase | (32) K_M_12 | 1.4 [37] | 1.4 | mM | ☒ | ☐ | ☒ | ☐ |
|  | (33) K_M_13 | 6.7×10^-2^ [38] | 6.7×10^-2^ | mM | ☒ | ☐ | ☒ | ☐ |
| (p)-AMPK kinase and phosphatase | (34) K_M_14 | 1.4 [37] | 1.4 | mM | ☒ | ☐ | ☒ | ☐ |
|  | (35) K_M_15 | 6.7×10^-2^ [38] | 6.7×10^-2^ | mM | ☒ | ☐ | ☒ | ☐ |
| ADP-(p)-AMPK kinase and phosphatase | (36) K_M_16 | 1.4 [37] | 1.4 | mM | ☒ | ☐ | ☒ | ☐ |
|  | (37) K_M_17 | 6.7×10^-2^ [38] | 6.7×10^-2^ | mM | ☒ | ☐ | ☒ | ☐ |
| AMP-(p)-AMPK kinase and phosphatase | (38) K_M_18 | 1.4 [37] | 1.4 | mM | ☒ | ☐ | ☒ | ☐ |
|  | (39) K_M_19 | 6.7×10^-2^ [38] | 6.7×10^-2^ | mM | ☒ | ☐ | ☒ | ☐ |
| AMPK kinase V_max_ | (40) V_maxKinase_ | 3.92×10^-2^ [37] | 5×10^-3^ | mM/s | ☒ | ☒ | ☒ | ☐ |
|  | (41) V_maxKinaseATP_ | 3.92×10^-2^ [37] | 5×10^-3^ | mM/s | ☒ | ☒ | ☒ | ☐ |
|  | (42) V_maxKinaseADP_ | 3.92×10^-2^ [37] | 7.5×10^-3^ | mM/s | ☒ | ☒ | ☒ | ☐ |
|  | (43) V_maxKinaseAMP_ | 3.92×10^-2^ [37] | 2.0×10^-2^ | mM/s | ☒ | ☒ | ☒ | ☐ |
| p-AMPK phosphatase V_max_ | (44) V_maxPPase_ | 1.1×10­^-1^ [38] | 1×10^-2^ | mM/s | ☒ | ☒ | ☒ | ☒ |
|  | (45) V_maxPPaseATP_ | 1.1×10­^-1^ [38] | 1×10^-2^ | mM/s | ☒ | ☒ | ☒ | ☒ |
|  | (46) V_maxPPaseADP_ | 1.1×10­^-1^ [38] | 1×10^-4^ | mM/s | ☒ | ☒ | ☒ | ☒ |
|  | (47) V_maxPPaseAMP_ | 1.1×10­^-1^ [38] | 1×10^-4^ | mM/s | ☒ | ☒ | ☒ | ☒ |
| *Pharmacological activator module*** | | | | | | | | |
| ZMP-(p)-AMPK kinase and phosphatase parameters | (48) V_maxKinaseZMP_ | NA | 2.0×10^-2^ | mM/s | ☐ | ☐ | ☐ | ☒ |
|  | (49) V_maxPPaseZMP_ | NA | 1×10^-4^ | mM/s | ☐ | ☐ | ☐ | ☒ |
|  | (50) K_M_20 | NA | 1.4 | mM | ☐ | ☐ | ☐ | ☒ |
|  | (51) K_M_21 | NA | 6.7×10^-2^ | mM | ☐ | ☐ | ☐ | ☒ |
| ZMP-AMPK binding | (52) k12f | NA | 1 [28] | 1/(mM×s) | ☒ | ☐ | ☐ | ☐ |
|  | (53) k12r | NA | 1.8×10^-2^ [28] | 1/s | ☒ | ☐ | ☐ | ☐ |
| ZMP-p-AMPK binding | (54) k13f | NA | 1 [28] | 1/(mM×s) | ☒ | ☐ | ☐ | ☐ |
|  | (55) k13r | NA | 1.8×10^-2^ [28] | 1/s | ☒ | ☐ | ☐ | ☐ |
| Infusion rate (AICAR to ZMP) | (56) k_AICAR_ | NA | 4×10^-4^ | 1/s | ☐ | ☐ | ☒ | ☐ |
| Degradation rate | (57) k_Act_ | NA | 3×10^-3^ | 1/s | ☐ | ☐ | ☒ | ☐ |
| *Additional parameters* | | | | | | | | |
| Muscle pH | pH | 7.0 [7,39] | 7.2 |  | ☒ | ☐ | ☐ | ☐ |
| AMPK turnover number | k_catAMPK_ | 30.6 [40] | 34.56 | /s | ☒ | ☒ | ☒ | ☐ |

*Ref, references; Calc, calculations; Calib, calibrations; Est, estimations.
**The initial pharmacological activator parameter values were based on ZMP, the values for Compound 991 are listed in Table F.

**Table E.** Allosteric activation potencies for simulations.

| **Simulation set** | **Value** | **Activator(s)** | **Fold-activation** | **Reference** |
| --- | --- | --- | --- | --- |
| Calibrated model | Base | AMP | 4 | [25,41] |
| MPSA, α1β2γ1 | Minimum | AMP | 1 | [31,42] |
|  | Base |  | 4 |  |
|  | Maximum |  | 13 |  |
| MPSA, α2β2γ3 | Minimum | AMP | 1 | [27] |
|  | Base |  | 1.54 |  |
|  | Maximum |  | 2 |  |
| Dose-response | Base | AMP | 4 | [25,41] |
|  |  | ZMP | 2 | [25,41] |
|  |  | C991 | 3.9 | [25] |

| Protocol | Initial concentrations | Kinetic parameter values | Reference |
| --- | --- | --- | --- |
| Stephens | See Table A | See Table D | n/a |
| Nielsen Sedentary | ATP_0_ = 8.24 mM AMP_0_ = 0.192 µM PCr_0_ = 27.8 mM TCr_0_ = 41.5 mM | k_rest_ and k_post_ = 1.4×10^-2^ /s  k_stim_ = 6.2×10^-2^ /s | [43] |
| Nielsen Trained | ATP_0_ = 7.86 mM AMP_0_ = 0.306 µM PCr_0_ = 23.3 mM  TCr_0_ = 37 mM | k_rest_ and k_post_ = 1.8×10^-2^ /s  k_stim_ = 5.2×10^-2^ /s |  |
| Gibala HIIE | ATP_0_ = 9.6 mM  PCr_0_ = 26.2 mM  TCr_0_ = 39.9 mM | k_rest_ and k_post_ = 1.7×10^-2^ /s  k_stim_ = 1.05×10^-1^ /s | [44] |
| ZMP |  | See Table D | n/a |
| Compound 991 |  | k12f = 1 /(mM×s)  k12r = 5.1×10^-4^ /s  k13f = 1 /(mM×s)  k13r = 5.1×10^-4^ /s  V_maxPPaseZMP_ = 2×10^-4^ mM/s  k_AICAR_ = 0;  k_Act_ = 0; | [25] |

Table F. Simulation-specific parameter values and initial conditions. Summary of the changes to the parameter values and initial concentrations from the original model to simulate the exercise protocols from different studies.

**Table G.** Activator properties for the dose-response analysis. ‘Baseline’ refers to the calibrated value for unphosphorylated and unbound AMPK.

| **Activator Name** | **Observed K_D_** | **Potency** | | | **Concentration Range** |
| --- | --- | --- | --- | --- | --- |
|  |  | *Increased Thr172 Phosphorylation* | *Decreased Thr172 Dephosphorylation* | *Allosteric Activation* |  |
| Compound  991 | 0.51 µM [25] | Assumed same as AMP | C991 V_max =_ 2×*V_maxPPaseAMP_* [25] | 3.9-fold*  [25] | 0.1-10 µM  [24] |
| ZMP | 18 µM [28] | Assumed same as AMP [41] | ZMP V_max =_ *V_maxPPaseAMP_* [41] | 2-fold  [41] | 500-2,000 µM [34] |
| ADP | 1.5 µM [16,27] | ⇧1.5-fold vs. baseline [42] | See Table D | No effect | 50-200 µM  [16] |
| AMP | 2.5 µM [16,27] | ⇧4-fold vs. baseline [42] | See Table D | 4-fold [42] | 0.5-5 µM  [16] |

*Average value from two AMPK isoforms, α1β2γ1 and α2β2γ1, for which data were available.

# Interpretation of Supplementary Figures S1 to S3

Supplementary Figures S1 to S3 present boxplots that summarize the distributions of parameter values, ratios of these values, and composite ratios for the “ADP-dominant” (green) and “AMP-dominant” (blue) models amongst the acceptable models from the MPSAs. The fraction of ADP-mediated control of AMPK activity was calculated using Equation 2 from the main text. Models for which the fraction of ADP control exceeded 0.8 were classified as “ADP-dominant” whereas those less than 0.2 were classified as “AMP-dominant”. Shifts of the blue and green boxplots relative to each other for a given parameter or ratio indicate that the quantity affects AMP or ADP dominance.

***Boxplots***. All values were plotted on the base-10 logarithmic scale. The black points represent the median, the box bounds are the first and third quartile values, the whiskers extend to the farthest point within 1.5× of the interquartile range, and the points beyond the whiskers are plotted as individual circles and are considered outliers. The horizontal red lines demarcate the bounds of the sampling ranges for the parameter values used in the MPSA. The location and width of the boxplots within the bounds indicate parameter regimes conducive to generating acceptable models.

***Variable labels***. Subscripts indicate the phosphorylation state (p = phosphorylated or 0 = dephosphorylated) and species bound to an adenine nucleotide are either preceded or succeeded by the identity of the bound nucleotide.

***Sample sizes***. The sample sizes for the MPSA boxplots were uneven because they depended on the number of acceptable models within the 50,000 simulations for each MPSA and the number of ADP- and AMP-dominant cases. The number of samples comprising each boxplot in each MPSA were as follows:

- Unconstrained: ADP, 61; AMP, 127
- α1β2γ1 K_D_: ADP, 2,289; AMP, 18
- α2β2γ3 K_D_: ADP, 1,390; AMP, 195

# Supplementary References

1. Westerblad H, Bruton JD, Katz A. Skeletal muscle: energy metabolism, fiber types, fatigue and adaptability. Exp Cell Res. 2010;316(18):3093–9.

2. Scott W, Stevens J, Binder-Macleod SA. Human skeletal muscle fiber type classifications. Phys Ther. 2001;81(11):1810–6.

3. Gollnick PD, Piehl K, Saltin B. Selective Glycogen Depletion Pattern in Human Muscle-Fibers After Exercise of Varying Intensity and at Varying Pedalling Rates. J Physiol. 1974;241(1):45-.

4. Vollestad NK, Tabata I, Medbo JI. Glycogen breakdown in different human muscle fibre types during exhaustive exercise of short duration. Acta Physiol Scand. 1992;144:135–41.

5. Karatzaferi C, De Haan A, Ferguson R, Van Mechelen W, Sargeant A. Phosphocreatine and ATP content in human single muscle fibres before and after maximum dynamic exercise. Pflügers Arch. 2001;442(3):467–74.

6. Sjogaard G, Saltin B. Extra- and intracellular water spaces in muscles of man at rest and with dynamic exercise. Am J Physiol. 1982;243(3):R271-80.

7. Vicini P, Kushmerick MJ. Cellular energetics analysis by a mathematical model of energy balance: estimation of parameters in human skeletal muscle. Am J Physiol Cell Physiol. 2000;279(1):C213-24.

8. Lambeth MJ, Kushmerick MJ. A computational model for glycogenolysis in skeletal muscle. Ann Biomed Eng. 2002;30(6):808–27.

9. Sahlin K, Broberg S. Adenine nucleotide depletion in human muscle during exercise: causality and significance of AMP deamination. Int J Sports Med. 1990;11(S 2):S62--S67.

10. Wilson DF. Regulation of metabolism: the rest-to-work transition in skeletal muscle. Am J Physiol Metab. 2015;309(9):E793–801.

11. Forbes SC, Paganini AT, Slade JM, Towse TF, Meyer RA. Phosphocreatine recovery kinetics following low and high intensity exercise in human triceps surae and rat posterior hindlimb muscles. Am J Physiol Integr Comp Physiol. 2009;

12. Horska A, Fishbein KW, Fleg JL, Spencer RGS. The relationship between creatine kinase kinetics and exercise intensity in human forearm is unchanged by age. Am J Physiol Metab. 2000;279(2):E333–9.

13. Birk JB, Wojtaszewski JFP. Predominant α2/β2/γ3 AMPK activation during exercise in human skeletal muscle. J Physiol. 2006;577(3):1021–32.

14. Kjøbsted R, Hingst JR, Fentz J, Foretz M, Sanz M-N, Pehmøller C, et al. AMPK in skeletal muscle function and metabolism. FASEB J. 2018;32(4):1741–77.

15. Calabrese MF, Rajamohan F, Harris MS, Caspers NL, Magyar R, Withka JM, et al. Structural basis for AMPK activation: natural and synthetic ligands regulate kinase activity from opposite poles by different molecular mechanisms. Structure. 2014;22(8):1161–72.

16. Xiao B, Sanders MJ, Underwood E, Heath R, Mayer F V, Carmena D, et al. Structure of mammalian AMPK and its regulation by ADP. Nature. 2011;472(7342):230–3.

17. Gu X, Yan Y, Novick SJ, Kovach A, Goswami D, Ke J, et al. Deconvoluting AMP-activated protein kinase (AMPK) adenine nucleotide binding and sensing. J Biol Chem. 2017;292(30):12653–66.

18. Willows R, Sanders MJ, Xiao B, Patel BR, Martin SR, Read J, et al. Phosphorylation of AMPK by upstream kinases is required for activity in mammalian cells. Biochem J. 2017;474(17):3059–73.

19. Mounier R, Théret M, Lantier L, Foretz M, Viollet B. Expanding roles for AMPK in skeletal muscle plasticity. Trends Endocrinol Metab. 2015;26(6):275–86.

20. Sakamoto K, Göransson O, Hardie DG, Alessi DR. Activity of LKB1 and AMPK-related kinases in skeletal muscle: effects of contraction, phenformin, and AICAR. Am J Physiol Endocrinol Metab. 2004;287(2):E310–7.

21. Steinberg GR, O’Neill HM, Dzamko NL, Galic S, Naim T, Koopman R, et al. Whole body deletion of AMP-activated protein kinase β2 reduces muscle AMPK activity and exercise capacity. J Biol Chem. 2010;285(48):37198–209.

22. Mollica JP, Oakhill JS, Lamb GD, Murphy RM. Are genuine changes in protein expression being overlooked? Reassessing Western blotting. Anal Biochem. 2009;386(2):270–5.

23. Sun Y, Connors KE, Yang D-Q. AICAR induces phosphorylation of AMPK in an ATM-dependent, LKB1-independent manner. Mol Cell Biochem. 2007 Oct;306(1–2):239–45.

24. Bultot L, Jensen TE, Lai Y-C, Madsen ALB, Collodet C, Kviklyte S, et al. Benzimidazole derivative small-molecule 991 enhances AMPK activity and glucose uptake induced by AICAR or contraction in skeletal muscle. Am J Physiol Endocrinol Metab. 2016 Oct;311(4):E706–19.

25. Xiao B, Sanders MJ, Carmena D, Bright NJ, Haire LF, Underwood E, et al. Structural basis of AMPK regulation by small molecule activators. Nat Commun. 2013;4:1–17.

26. Kim KA, Spencer SL, Albeck JG, Burke JM, Sorger PK, Gaudet S, et al. Systematic calibration of a cell signaling network model. BMC Bioinformatics. 2010/04/27. 2010;11:202.

27. Rajamohan F, Reyes AR, Frisbie RK, Hoth LR, Sahasrabudhe P, Magyar R, et al. Probing the enzyme kinetics, allosteric modulation and activation of α1-and α2-subunit-containing AMP-activated protein kinase (AMPK) heterotrimeric complexes by pharmacological and physiological activators. Biochem J. 2016;473(5):581–92.

28. Saiu P. Structural and functional studies on nucleotide binding to AMP-activated protein kinase. University College London; 2010.

29. Neves SR. Obtaining and estimating kinetic parameters from the literature. Sci Signal. 2011 Sep 20 [cited 2015 Feb 13];4(191):tr8.

30. Stephens TJ, Chen Z-P, Canny BJ, Michell BJ, Kemp BE, McConell GK. Progressive increase in human skeletal muscle AMPKalpha2 activity and ACC phosphorylation during exercise. Am J Physiol Endocrinol Metab. 2002;282(3):E688–94.

31. Gowans GJ, Hawley SA, Ross FA, Hardie DG. AMP is a true physiological regulator of AMP-activated protein kinase by both allosteric activation and enhancing net phosphorylation. Cell Metab. 2013 Oct 1 [cited 2015 Dec 8];18(4):556–66.

32. Sriwijitkamol A, Coletta DK, Wajcberg E, Balbontin GB, Reyna SM, Barrientes J, et al. Effect of acute exercise on AMPK signaling in skeletal muscle of subjects with type 2 diabetes: A time-course and dose-response study. Diabetes. 2007;56(3):836–48.

33. Fujii N, Hayashi T, Hirshman MF, Smith JT, Habinowski S a, Kaijser L, et al. Exercise induces isoform-specific increase in 5’AMP-activated protein kinase activity in human skeletal muscle. Biochem Biophys Res Commun. 2000;273:1150–5.

34. Merrill GF, Kurth EJ, Hardie DG, Winder WW. AICA riboside increases AMP-activated protein kinase, fatty acid oxidation, and glucose uptake in rat muscle. Am J Physiol. 1997 Dec;273(6 Pt 1):E1107-12.

35. Suter M, Riek U, Tuerk R, Schlattner U, Wallimann T, Neumann D. Dissecting the role of 5’-AMP for allosteric stimulation, activation, and deactivation of AMP-activated protein kinase. J Biol Chem. 2006 Oct 27 [cited 2015 Dec 11];281(43):32207–16.

36. Malucelli E, Lodi R, Martinuzzi A, Tonon C, Barbiroli B, Iotti S. Free Mg2+ concentration in the calf muscle of glycogen phosphorylase and phosphofructokinase deficiency patients assessed in different metabolic conditions by 31P MRS. Dyn Med. 2005;4:7.

37. Lizcano JM, Göransson O, Toth R, Deak M, Morrice NA, Boudeau J, et al. LKB1 is a master kinase that activates 13 kinases of the AMPK subfamily, including MARK/PAR-1. EMBO J. 2004 Feb;23(4):833–43.

38. Marley AE, Sullivan JE, Carling D, Abbott WM, Smith GJ, Taylor IW, et al. Biochemical characterization and deletion analysis of recombinant human protein phosphatase 2C alpha. Biochem J. 1996 Dec;320 (Pt 3):801–6.

39. Veech RL, Lawson JW, Cornell NW, Krebs H a. Cytosolic phosphorylation potential. J Biol Chem. 1979;254(14):6538–47.

40. Michell BJ, Stapleton D, Mitchelhill KI, House CM, Katsis F, Witters LA, et al. Isoform-specific purification and substrate specificity of the 5’-AMP-activated protein kinase. J Biol Chem. 1996 Nov;271(45):28445–50.

41. Corton JM, Gillespie JG, Hawley S a, Hardie DG. 5-aminoimidazole-4-carboxamide ribonucleoside. A specific method for activating AMP-activated protein kinase in intact cells? Eur J Biochem. 1995;229(2):558–65.

42. Ross FA, Jensen TE, Hardie DG. Differential regulation by AMP and ADP of AMPK complexes containing different γ subunit isoforms. Biochem J. 2016;473(2):189–99.

43. Nielsen JN, Mustard KJW, Graham D a, Yu H, MacDonald CS, Pilegaard H, et al. 5’-AMP-activated protein kinase activity and subunit expression in exercise-trained human skeletal muscle. J Appl Physiol. 2003;94:631–41.

44. Gibala MJ, Mcgee SL, Garnham AP, Howlett KF, Snow RJ, Hargreaves M. Brief intense interval exercise activates AMPK and p38 MAPK signaling and increases the expression of PGC-1 alpha in human skeletal muscle. J Appl Physiol. 2009 Mar;106(3):929–34.
