# Supplementary material for: ADP is the dominant controller of AMP-activated protein kinase activity dynamics in skeletal muscle during exercise
Source: PLoS Comput Biol. 2020 Jul 30;16(7):e1008079. doi: 10.1371/journal.pcbi.1008079 (PMC7433884; doi:10.1371/journal.pcbi.1008079)
Supplement: S1 Fig — A. Effects of AXP-(p)-AMPK binding constants on AMP- versus ADP-dominant control of AMPK activity. i) Boxplots summarize the individual parameter values representing the forward (kf) and reverse (kr) rate constants. ii) Boxplots summarize the dissociation constants (KD) for each AXP-(p)-AMPK reaction, calculated as kr/kf from each simulation. iii) Boxplots summarize the ratios of all possible pairs of KD values. B. Effects of parameters determining the enhancement of AMPK phosphorylation on AMP- versus ADP-dominant control of AMPK activity in the Unconstrained MPSA. i) Boxplots summarize the Michaelis constants (KM) and maximum enzyme velocities (Vmax) for the AMPK kinase and phosphatase. ii) Boxplots summarize the catalytic efficiencies of the AMPK kinase and phosphatase, which was calculated as Vmax/KM for each simulation. iii) Boxplots summarize the compound ratios of kinase and phosphatase catalytic efficiencies. C. Effects of KeqADK, AMP and ADP concentrations, and allostery on AMP- versus ADP-dominant control of AMPK activity in the Unconstrained MPSA. Panels from left to right: i) Boxplots summarize the KeqADK values. ii) and iii) Boxplots summarize the maximum predicted concentrations of ADP (ii) and AMP (iii) during simulated exercise. The dashed-grey horizontal lines represent the reported maximum levels of ADP or AMP in moderate-intensity exercise studies. iv) Boxplots summarize the ratios of the maximum concentrations of ADP and AMP during simulated exercise. v) Boxplots summarize the magnitudes of allosteric activation of the AMP-p-AMPK complex. (PDF) [file pcbi.1008079.s004.pdf]

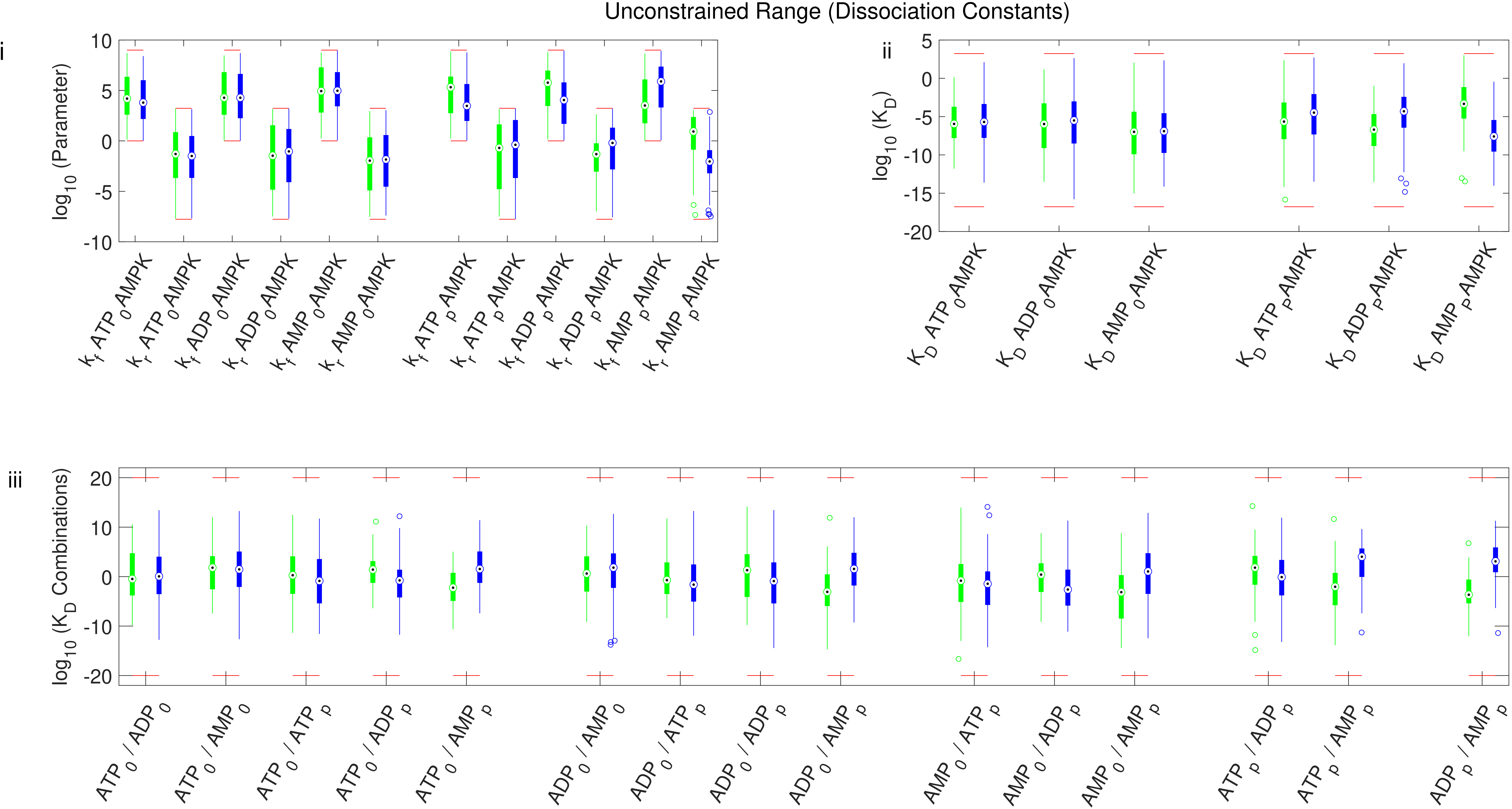

Unconstrained Range (Dissociation Constants)
i
log10 (Parameter)
10
5
0
-5
-10
kf ATP 0AMPK
kr ATP 0AMPK
kf ADP 0AMPK
kr ADP 0AMPK
kf AMP 0AMPK
kr AMP 0AMPK
kf ATP pAMPK
kr ATP pAMPK
kf ADP pAMPK
kr ADP pAMPK
kf AMP pAMPK
kr AMP pAMPK
ii
log10 (KD)
5
0
-5
-10
-15
-20
KD ATP 0AMPK
KD ADP 0AMPK
KD AMP 0AMPK
KD ATP pAMPK
KD ADP pAMPK
KD AMP pAMPK
iii
log10 (KD Combinations)
20
10
0
-10
-20
ATP 0 / ADP 0
ATP 0 / AMP 0
ATP 0 / ATP p
ATP 0 / ADP p
ATP 0 / AMP p
ADP 0 / AMP 0
ADP 0 / ATP p
ADP 0 / ADP p
ADP 0 / AMP p
AMP 0 / ATP p
AMP 0 / ADP p
AMP 0 / AMP p
ATP p / ADP p
ATP p / AMP p
ADP p / AMP p

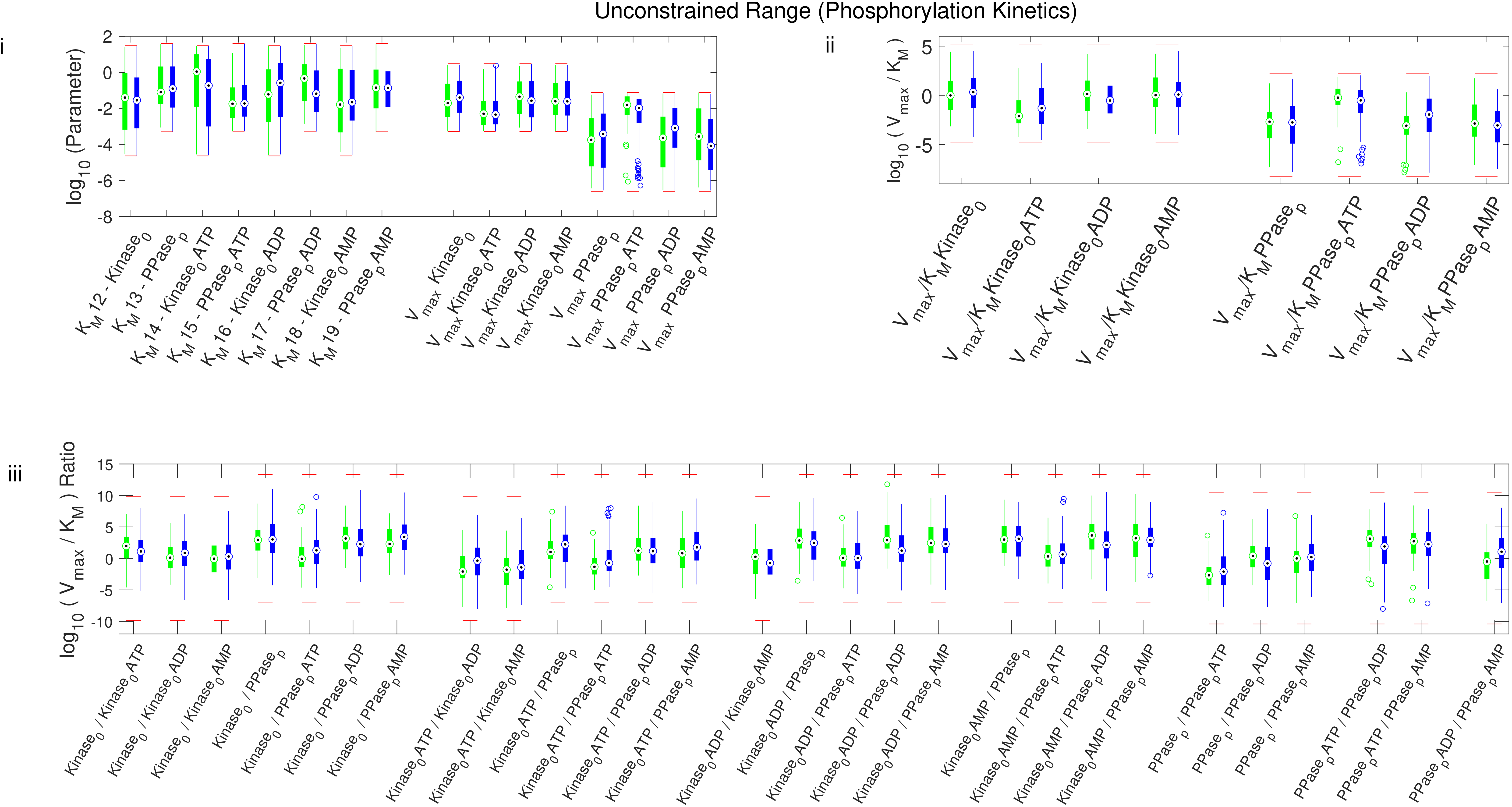

Unconstrained Range (Phosphorylation Kinetics)
i
log10 (Parameter)
K_M 12 - Kinase_0
K_M 13 - PPase_p
K_M 14 - Kinase_0 ATP
K_M 15 - PPase_p ATP
K_M 16 - Kinase_0 ADP
K_M 17 - PPase_p ADP
K_M 18 - Kinase_0 AMP
K_M 19 - PPase_p AMP
V_max Kinase_0
V_max Kinase_0 ATP
V_max Kinase_0 ADP
V_max Kinase_0 AMP
V_max PPase_p
V_max PPase_p ATP
V_max PPase_p ADP
V_max PPase_p AMP
ii
log10 (V_max / K_M)
V_max/K_M Kinase_0
V_max/K_M Kinase_0 ATP
V_max/K_M Kinase_0 ADP
V_max/K_M Kinase_0 AMP
V_max/K_M PPase_p
V_max/K_M PPase_p ATP
V_max/K_M PPase_p ADP
V_max/K_M PPase_p AMP
iii
log10 (V_max / K_M) Ratio
Kinase_0 / Kinase_0 ATP
Kinase_0 / Kinase_0 ADP
Kinase_0 / Kinase_0 AMP
Kinase_0 / PPase_p
Kinase_0 / PPase_p ATP
Kinase_0 / PPase_p ADP
Kinase_0 / PPase_p AMP
Kinase_0 ATP / Kinase_0 ADP
Kinase_0 ATP / Kinase_0 AMP
Kinase_0 ATP / PPase_p
Kinase_0 ATP / PPase_p ATP
Kinase_0 ATP / PPase_p ADP
Kinase_0 ATP / PPase_p AMP
Kinase_0 ADP / Kinase_0 AMP
Kinase_0 ADP / PPase_p
Kinase_0 ADP / PPase_p ATP
Kinase_0 ADP / PPase_p ADP
Kinase_0 ADP / PPase_p AMP
Kinase_0 AMP / PPase_p
Kinase_0 AMP / PPase_p ATP
Kinase_0 AMP / PPase_p ADP
Kinase_0 AMP / PPase_p AMP
PPase_p / PPase_p ATP
PPase_p / PPase_p ADP
PPase_p / PPase_p AMP
PPase_p ATP / PPase_p ADP
PPase_p ATP / PPase_p AMP
PPase_p ADP / PPase_p AMP

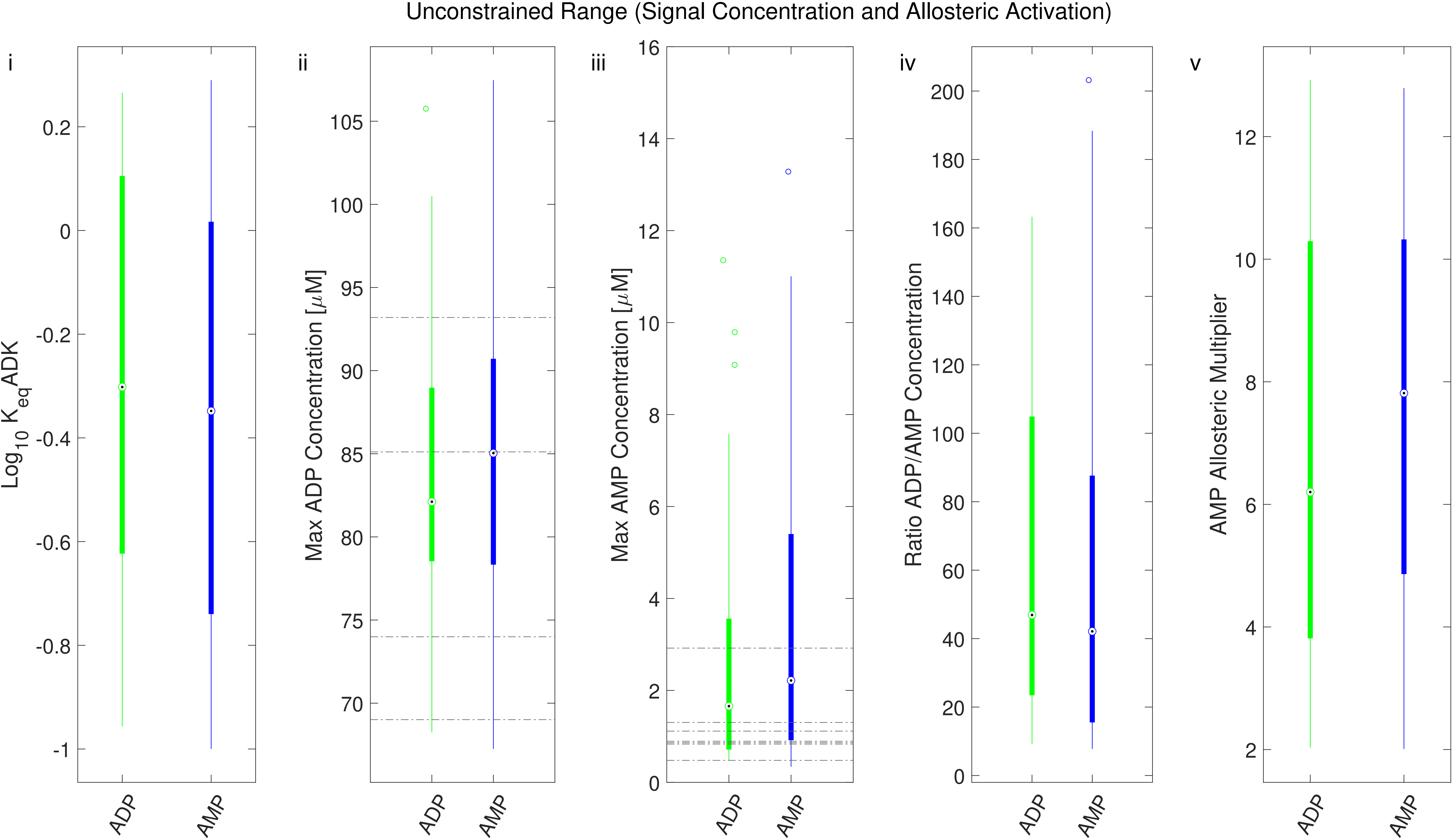
Unconstrained Range (Signal Concentration and Allosteric Activation)
i
ii
iii
iv
v
Log10 Keq ADK
Max ADP Concentration [μM]
Max AMP Concentration [μM]
Ratio ADP/AMP Concentration
AMP Allosteric Multiplier
ADP
AMP
