# Supplementary figures and images for: ADP is the dominant controller of AMP-activated protein kinase activity dynamics in skeletal muscle during exercise
Source: PLoS Comput Biol. 2020 Jul 30;16(7):e1008079. doi: 10.1371/journal.pcbi.1008079 (PMC7433884; doi:10.1371/journal.pcbi.1008079)

$\alpha_1\beta_2\gamma_1$  1.1x (Dissociation Constants)

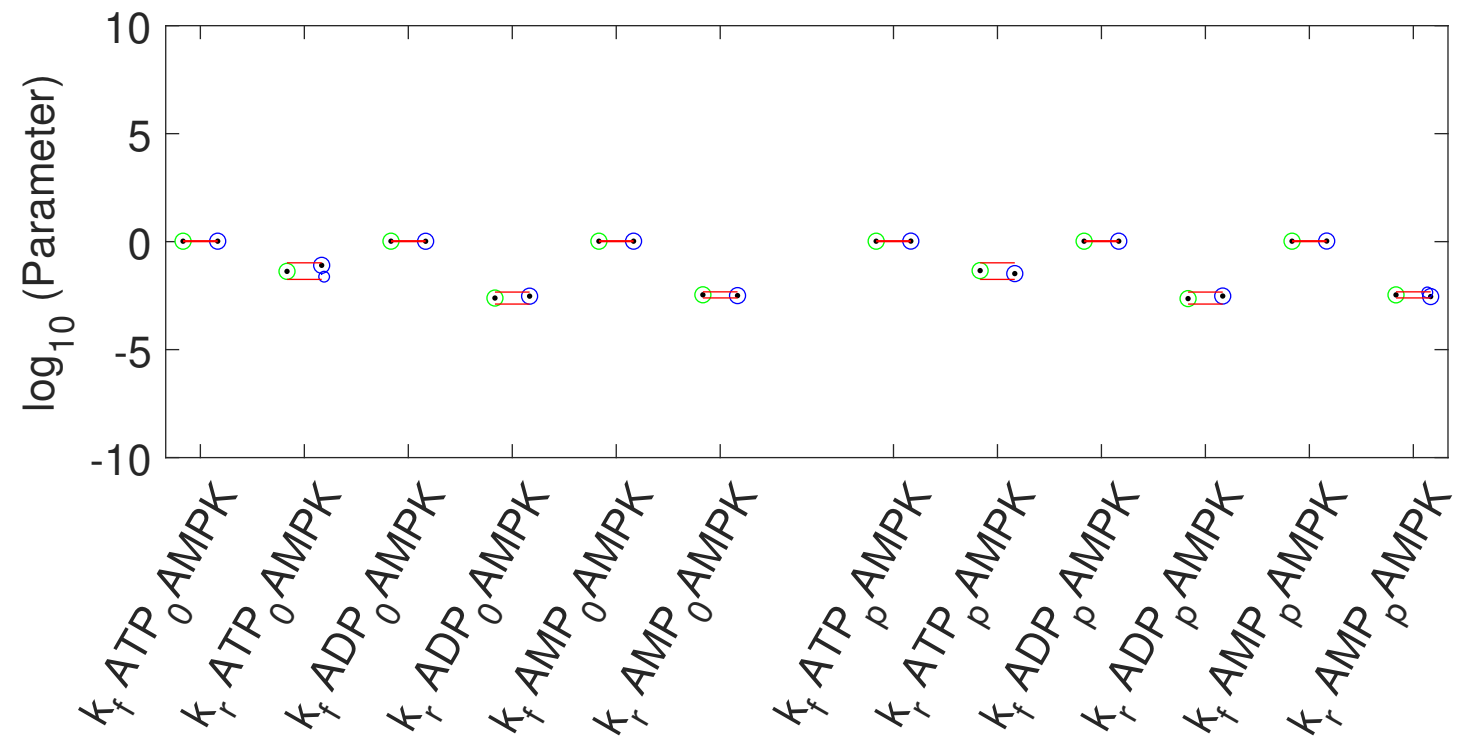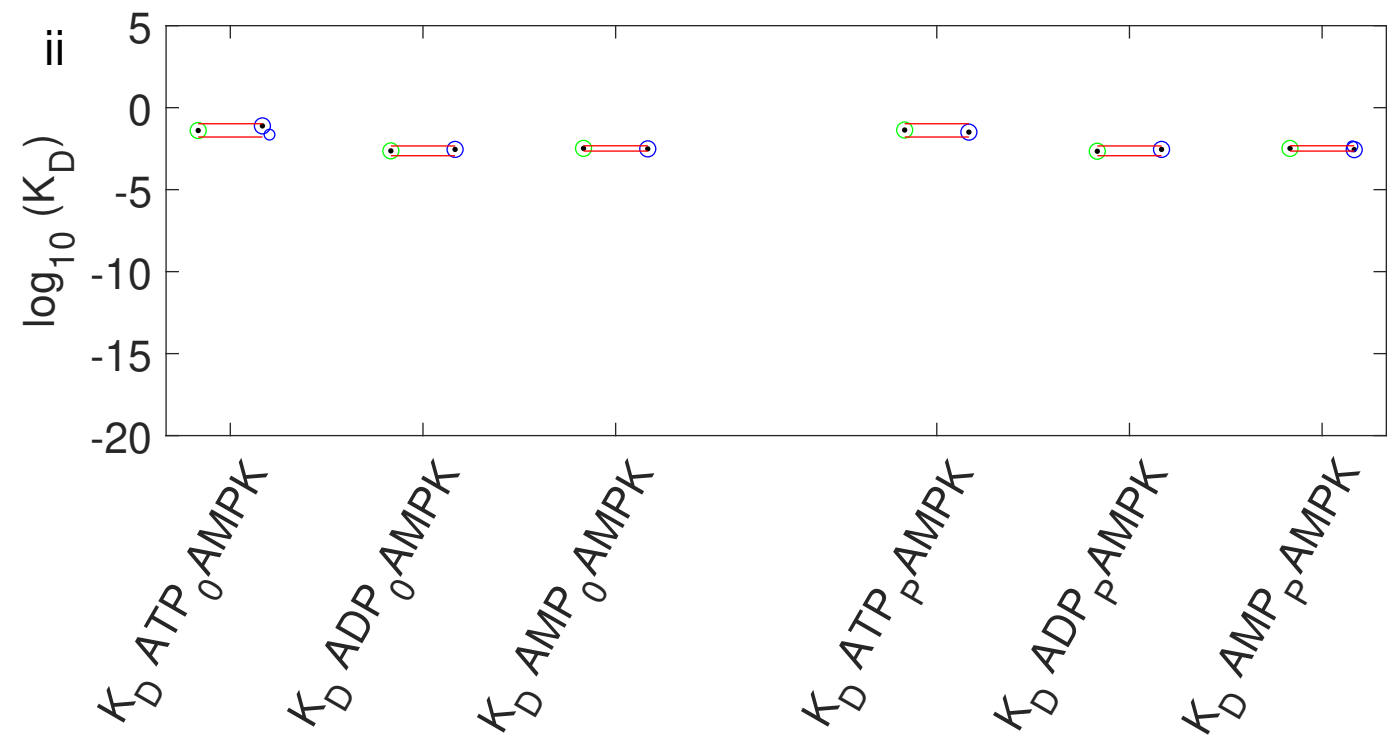

iii

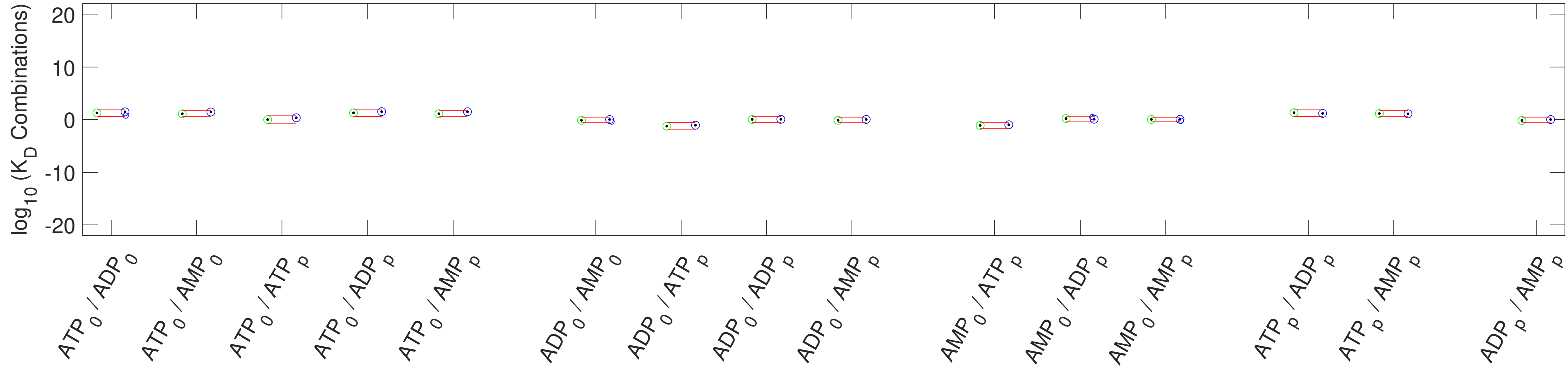

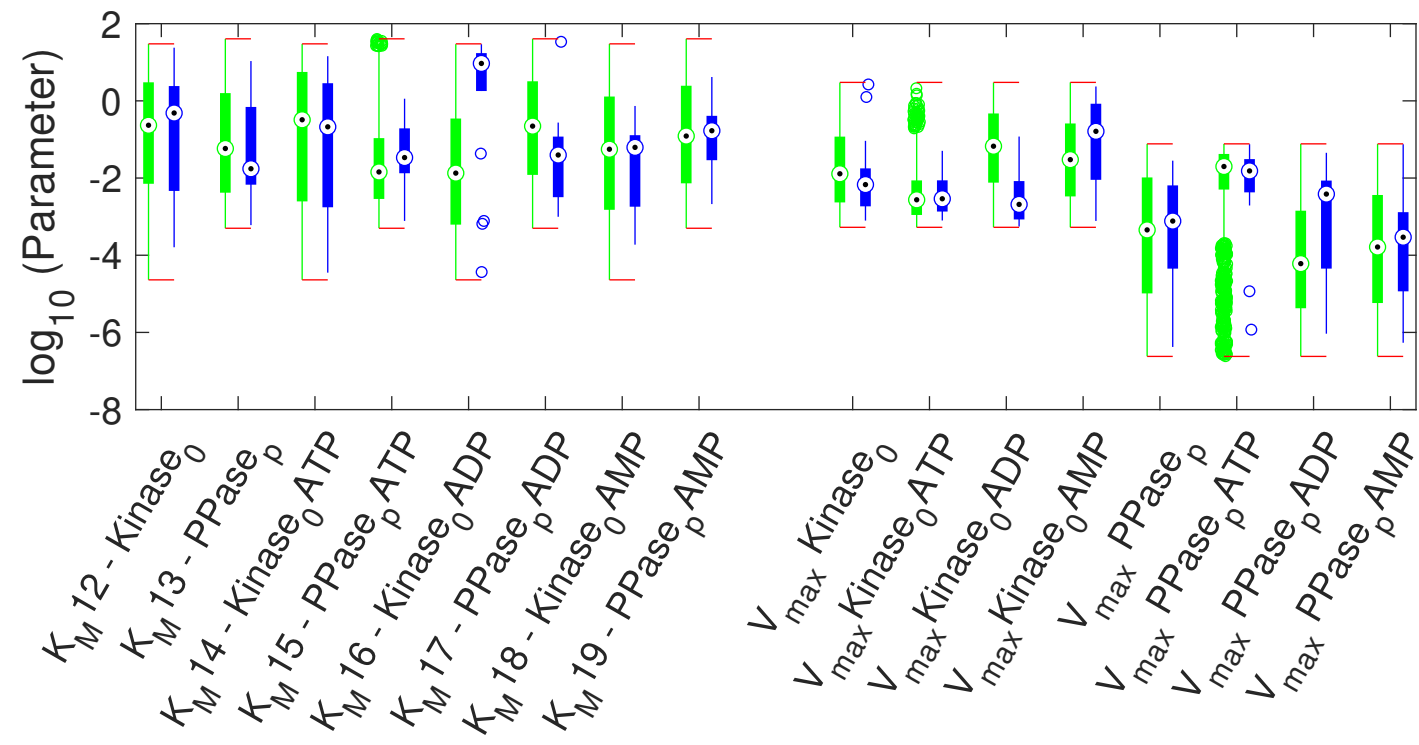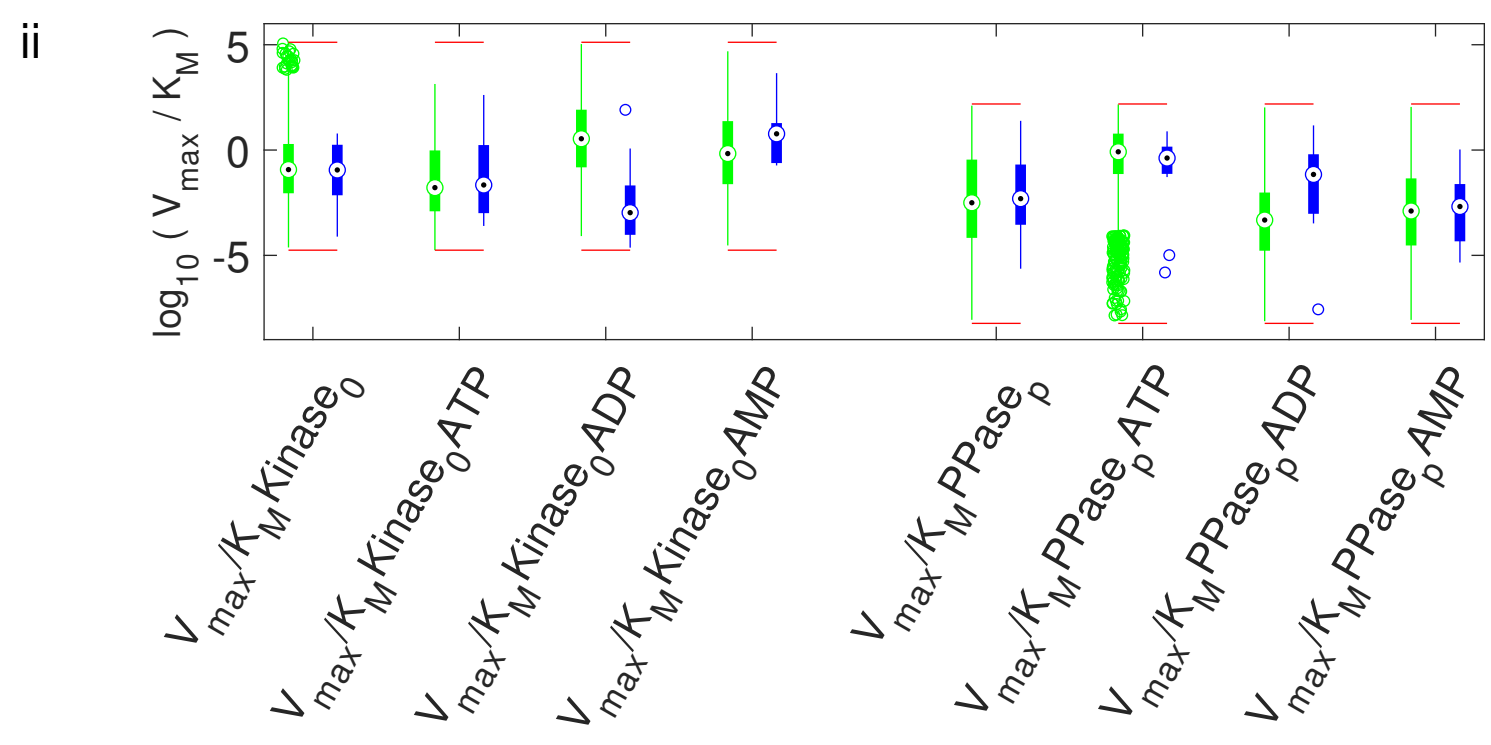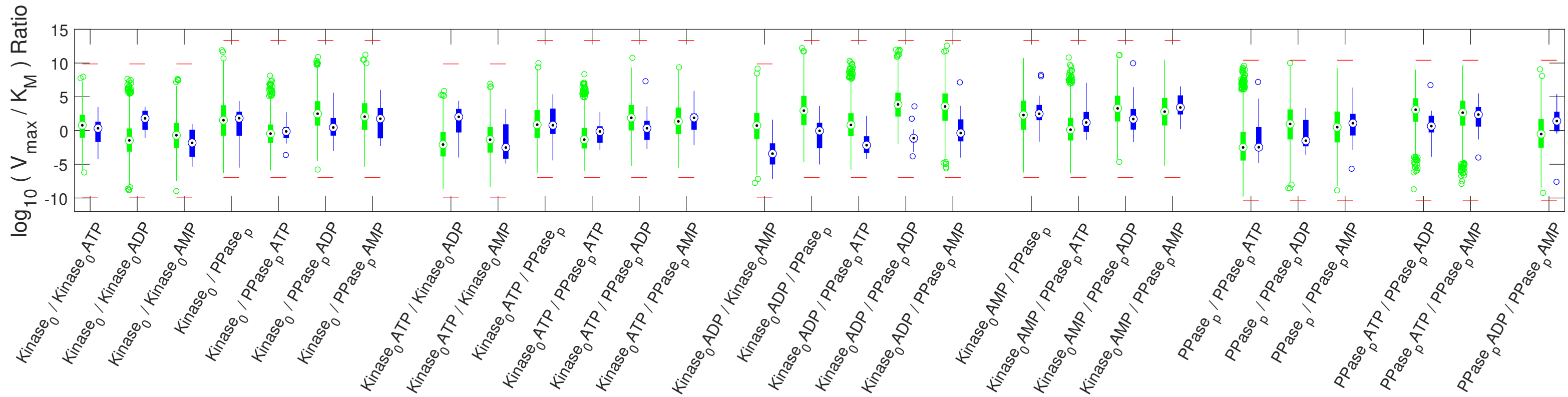

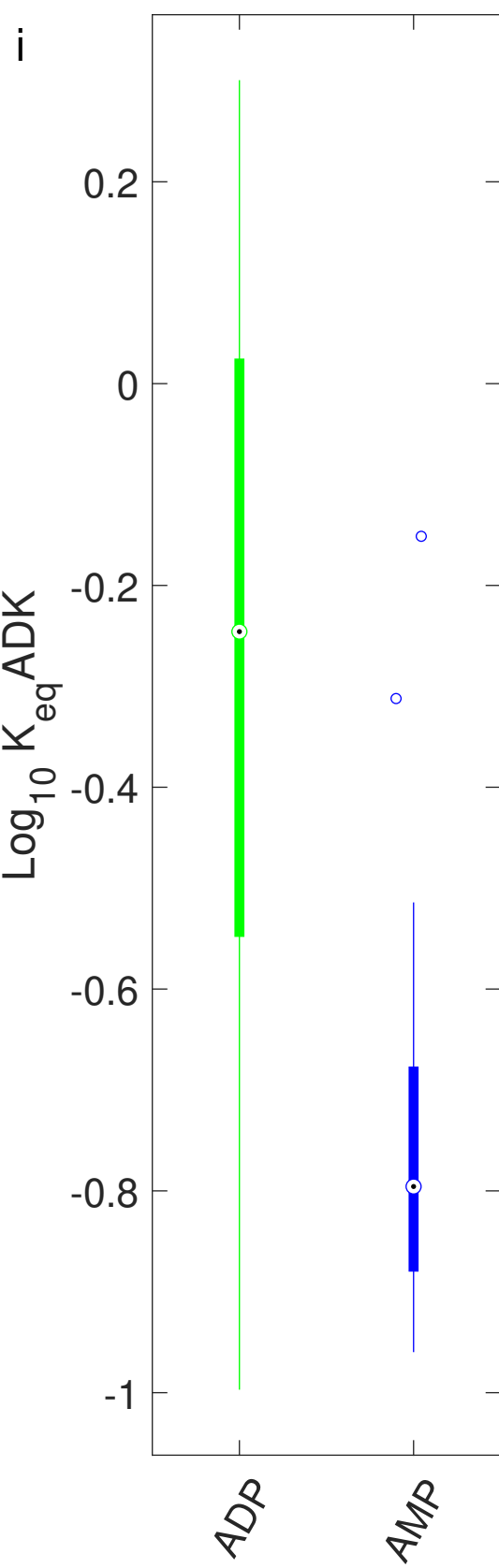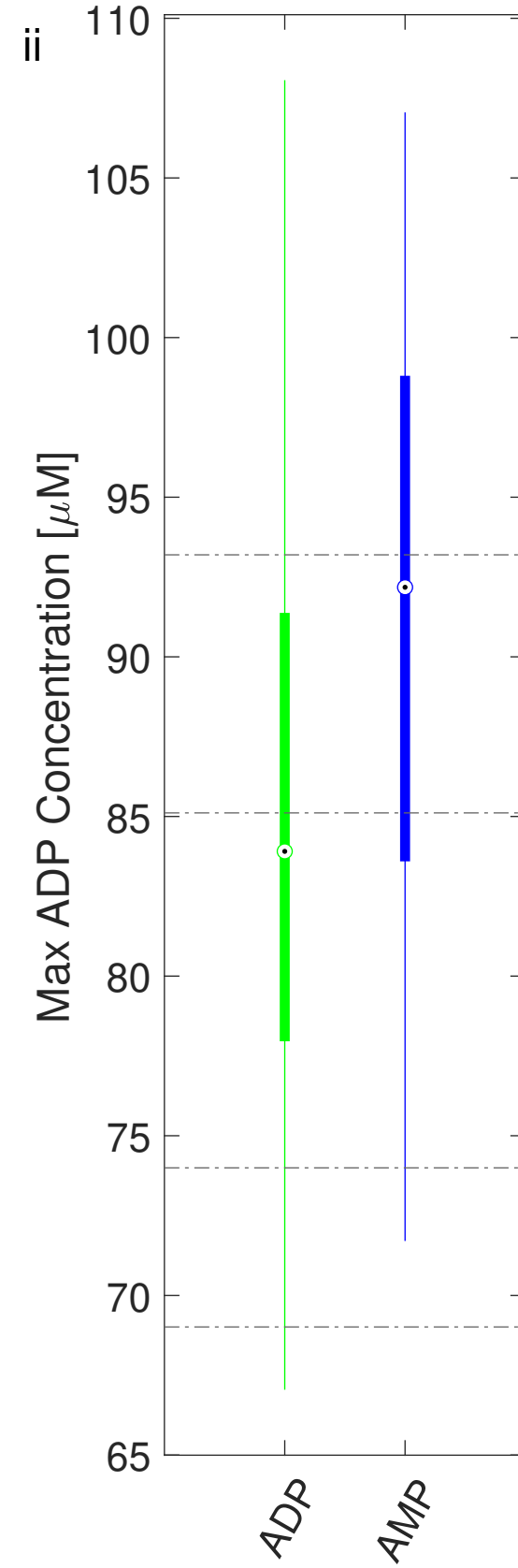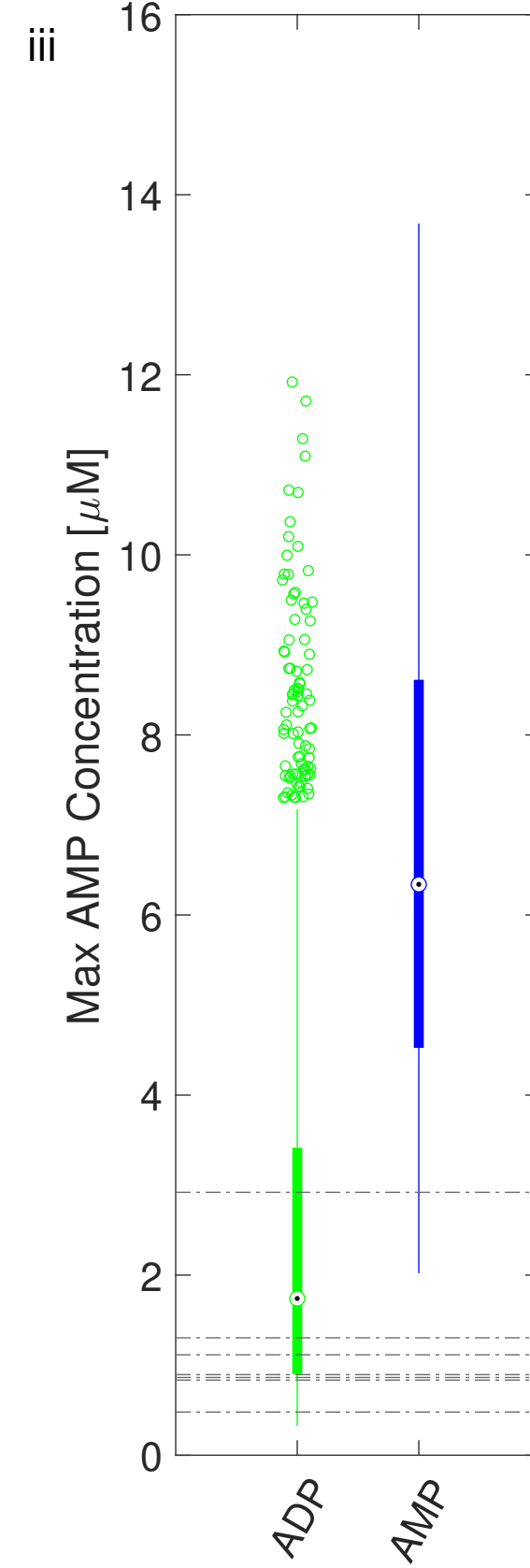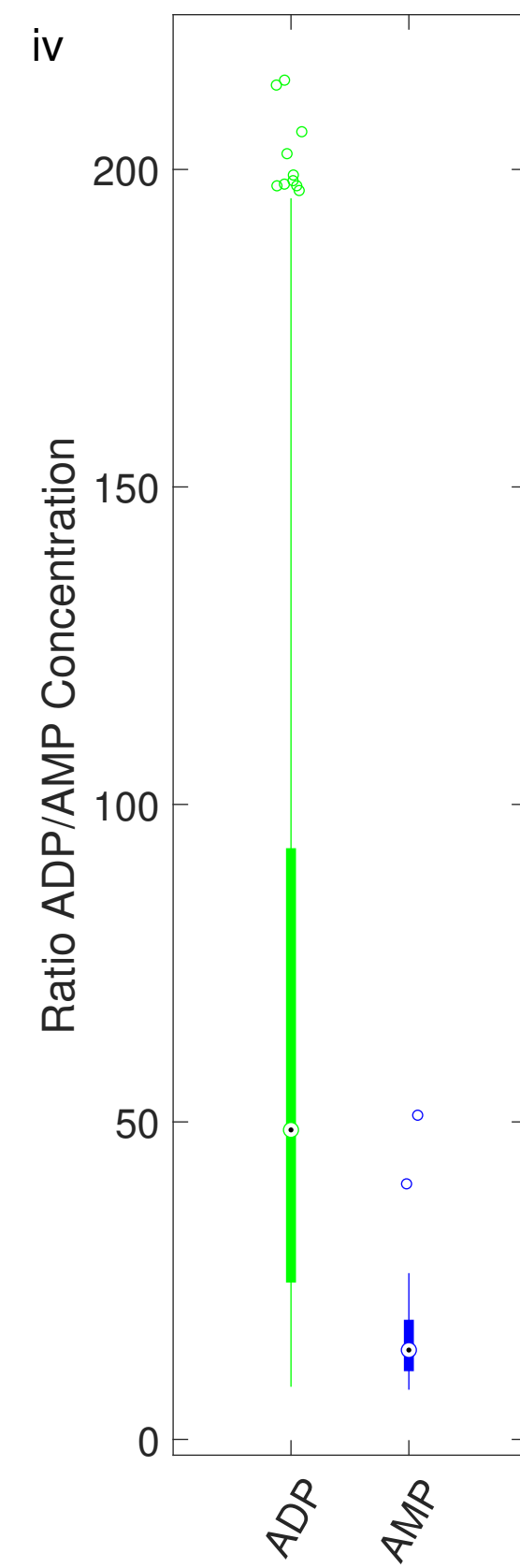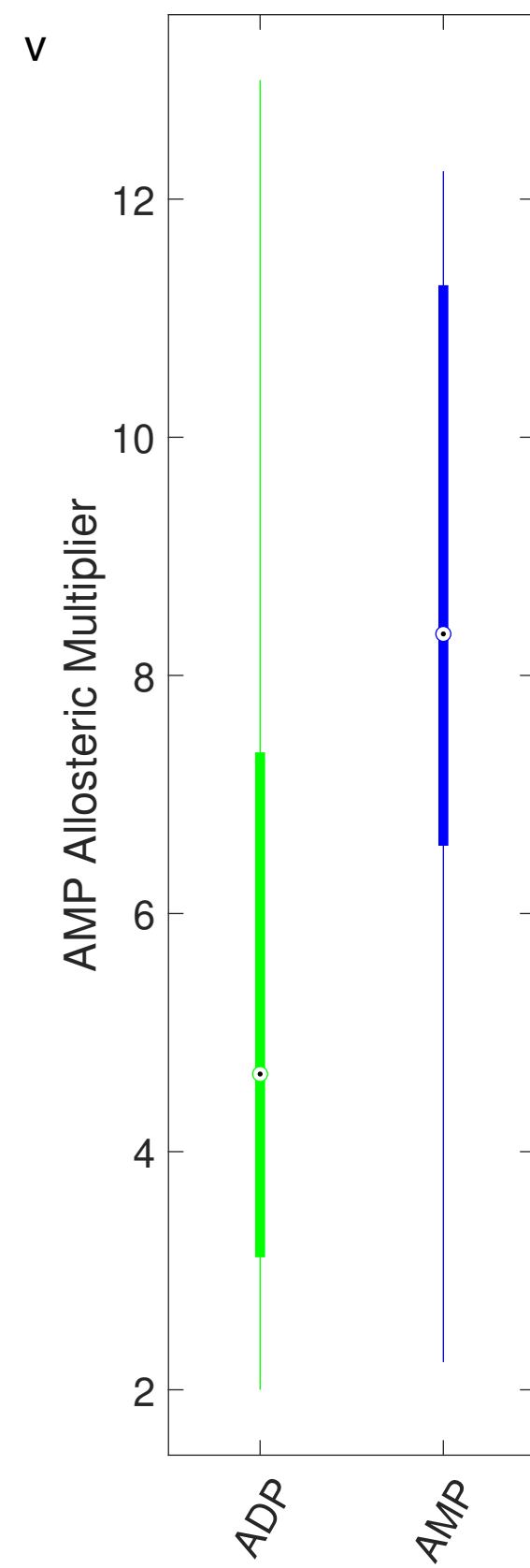

Supplement: S2 Fig — A. Effects of AXP-(p)-AMPK binding constants on AMP- versus ADP-dominant control of AMPK activity. i) Boxplots summarize the individual parameter values representing the forward (kf) and reverse (kr) rate constants. ii) Boxplots summarize the dissociation constants (KD) for each AXP-(p)-AMPK reaction, calculated as kr/kf from each simulation. iii) Boxplots summarize the ratios of all possible pairs of KD values. B. Effects of parameters determining the enhancement of AMPK phosphorylation on AMP- versus ADP-dominant control of AMPK activity in the α1β2γ1 KD MPSA. i) Boxplots summarize the Michaelis constants (KM) and maximum enzyme velocities (Vmax) for the AMPK kinase and phosphatase. ii) Boxplots summarize the catalytic efficiencies of the AMPK kinase and phosphatase, which was calculated as Vmax/KM for each simulation. Bottom panel: Boxplots summarize the compound ratios of kinase and phosphatase catalytic efficiencies. C. Effects of KeqADK, AMP and ADP concentrations, and allostery on AMP- versus ADP-dominant control of AMPK activity in the α1β2γ1 KD MPSA. Panels from left to right: i) Boxplots summarize the KeqADK values. ii) and iii) Boxplots summarize the maximum predicted concentrations of ADP (ii) and AMP (iii) during simulated exercise. The dashed-grey horizontal lines represent the reported maximum levels of ADP or AMP in moderate-intensity exercise studies. iv) Boxplots summarize the ratios of the maximum concentrations of ADP and AMP during simulated exercise. v) Boxplots summarize the magnitudes of allosteric activation of the AMP-p-AMPK complex. (PDF) [file pcbi.1008079.s005.pdf]

$\alpha 2 \beta 2 \gamma 3$  1.1x (Dissociation Constants)

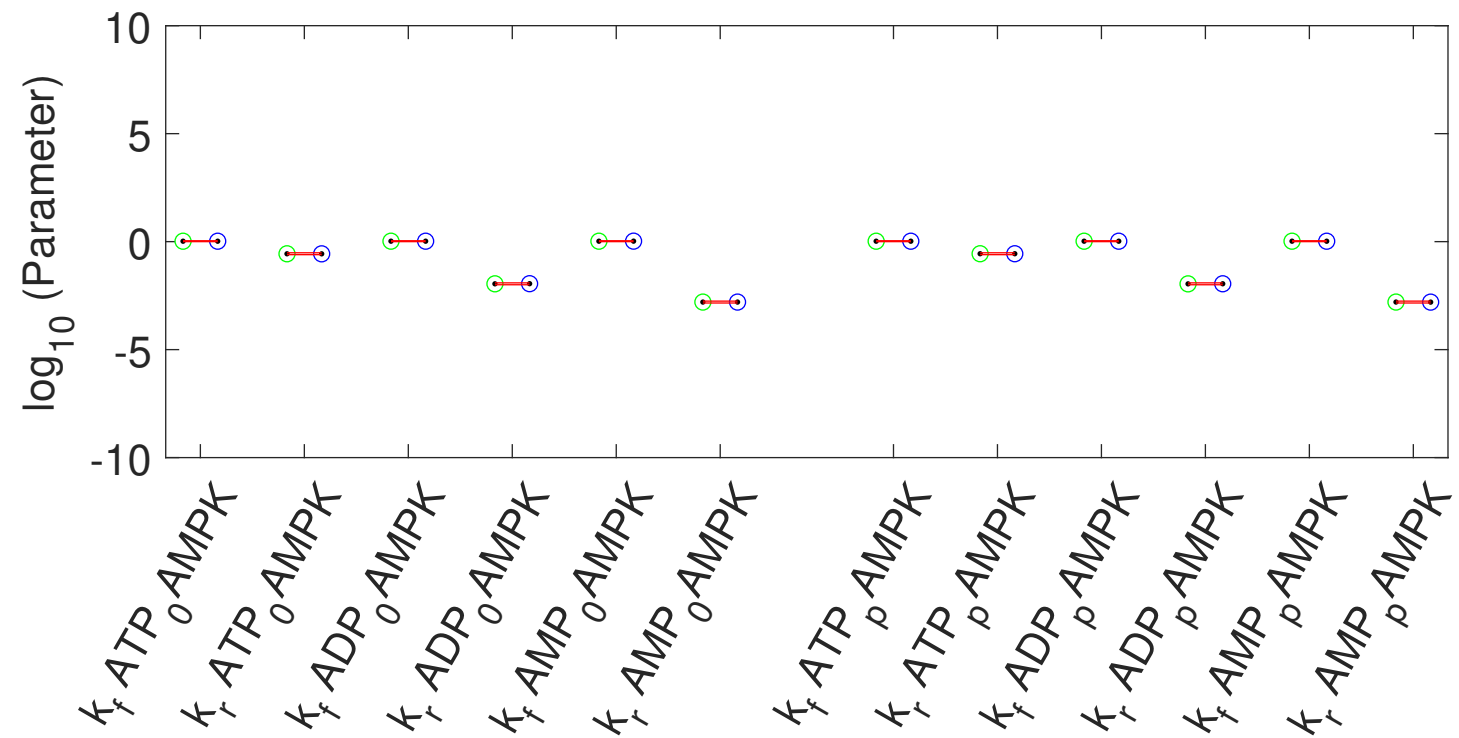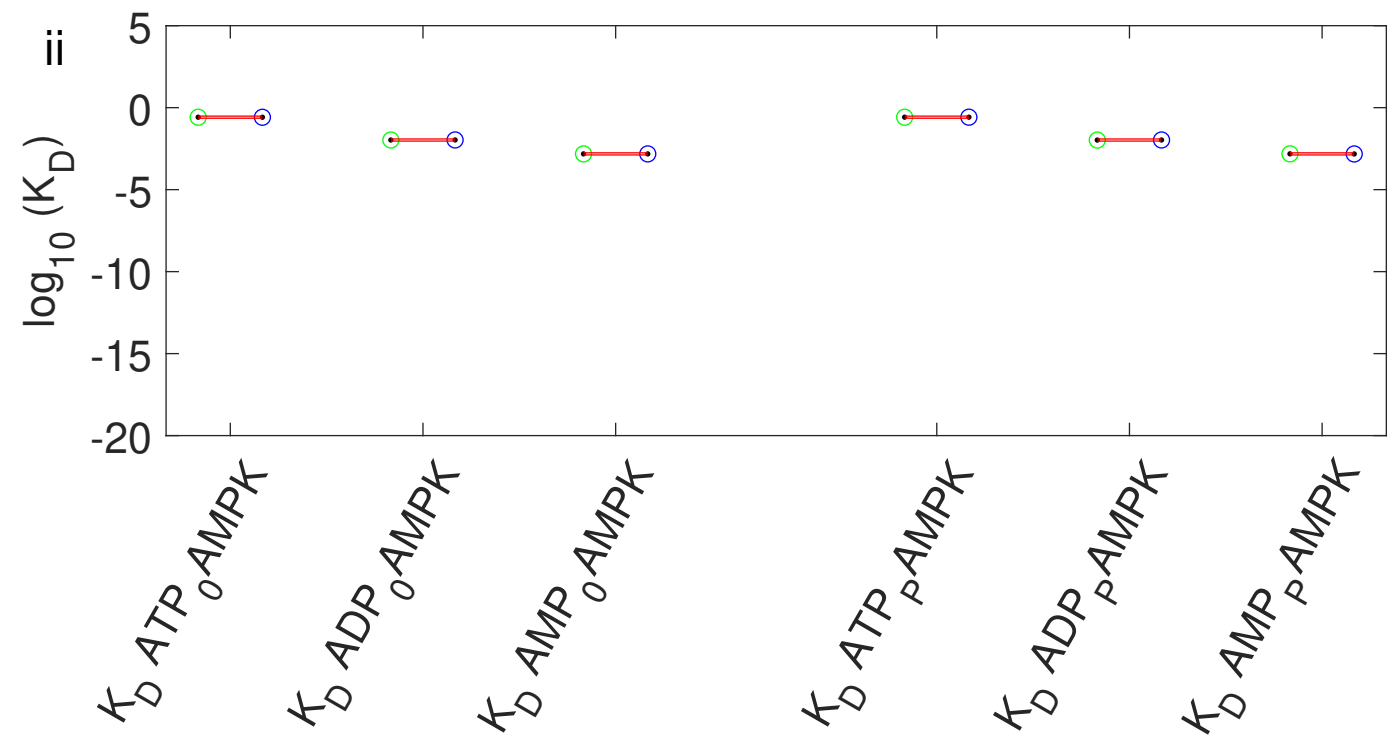

iii

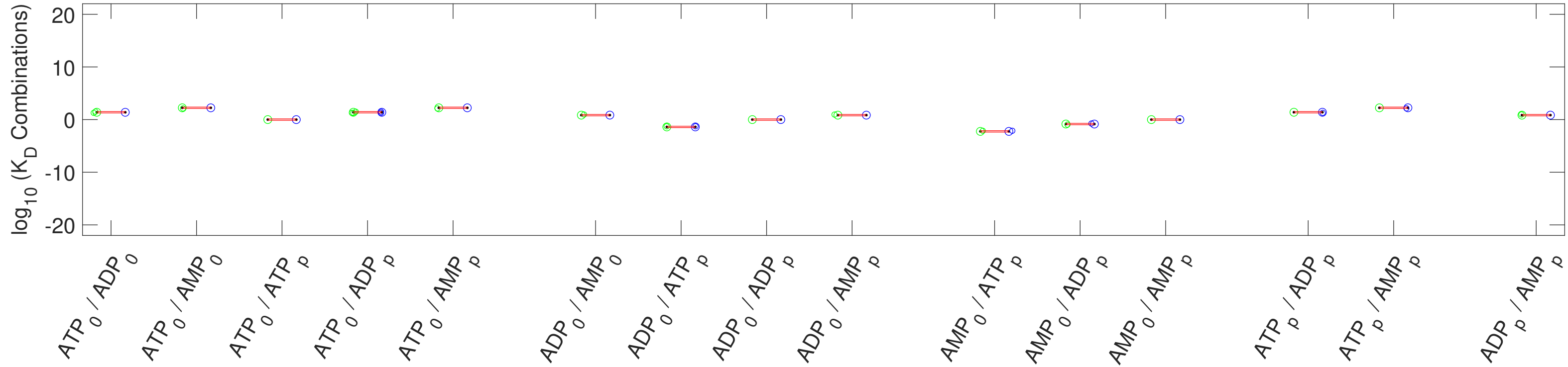

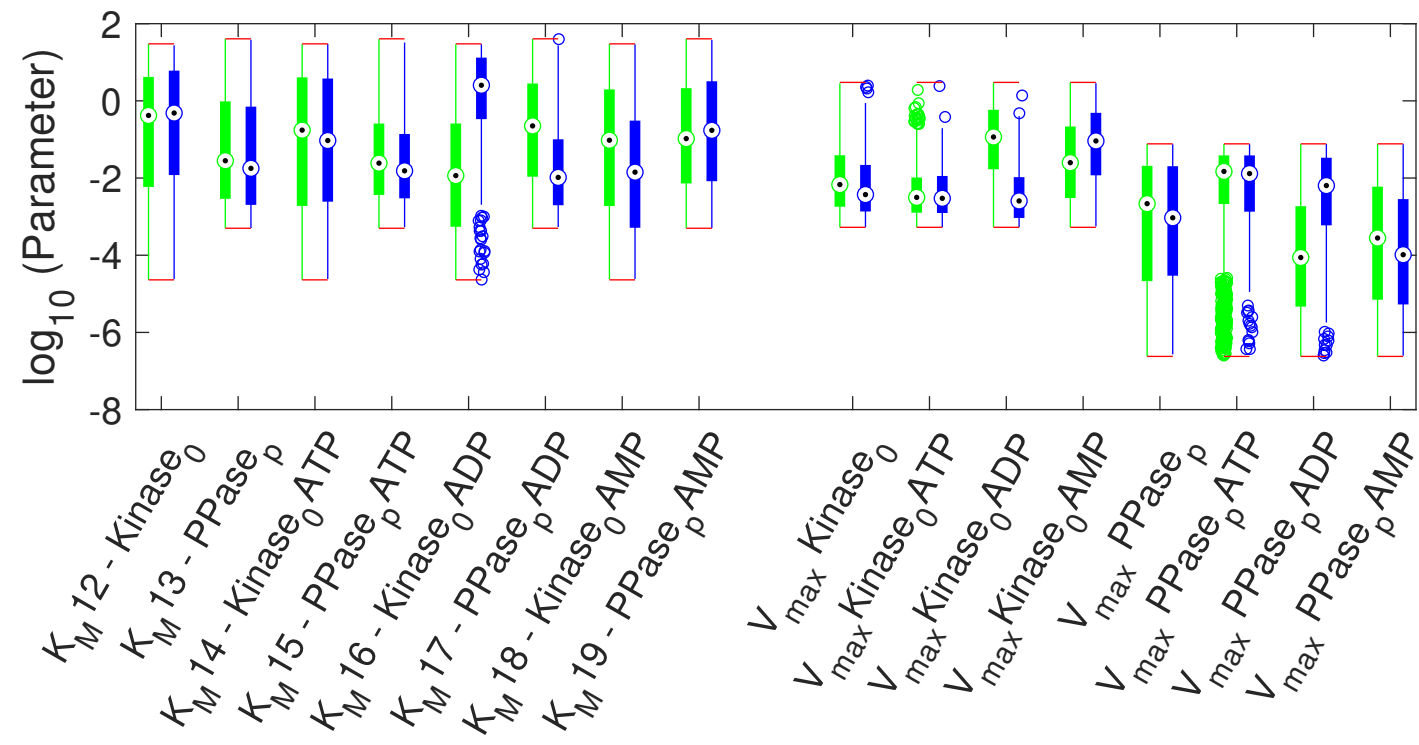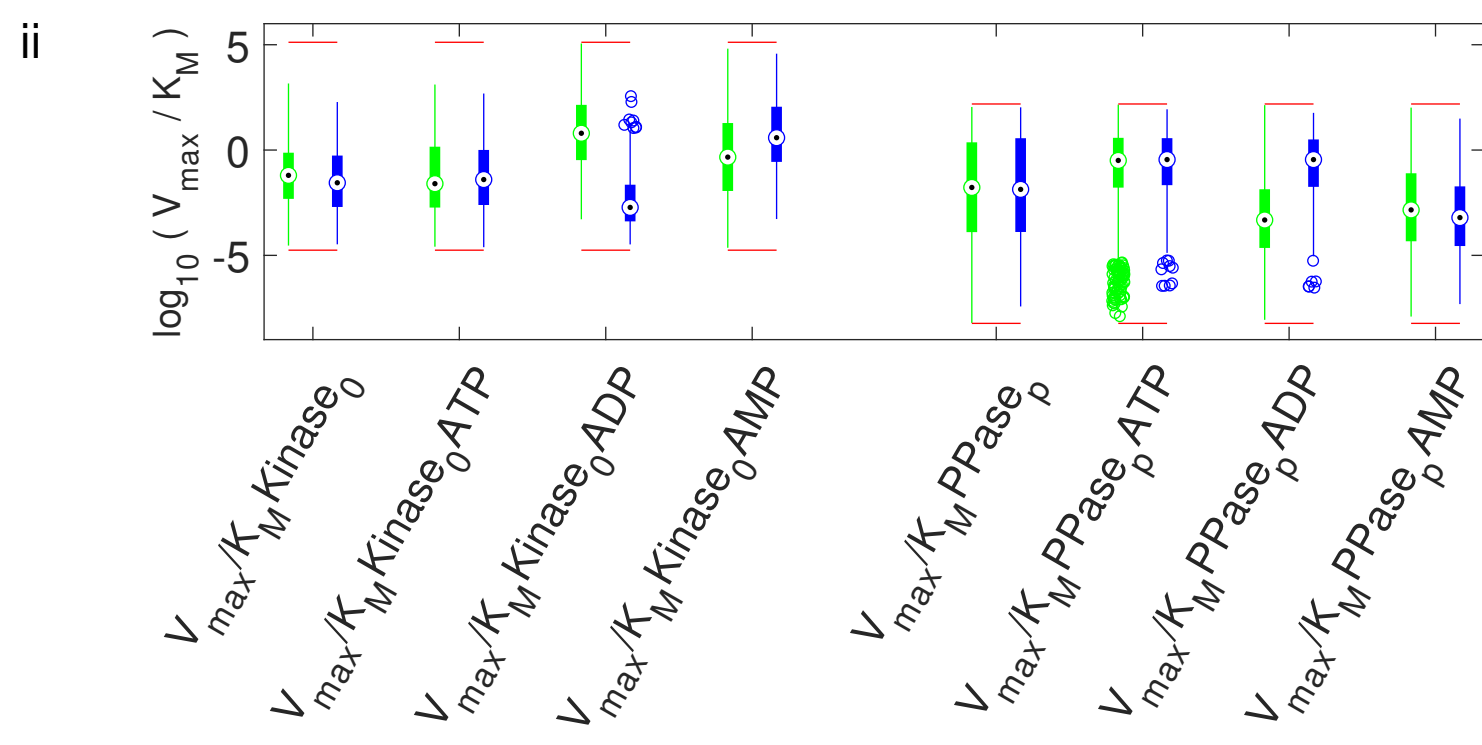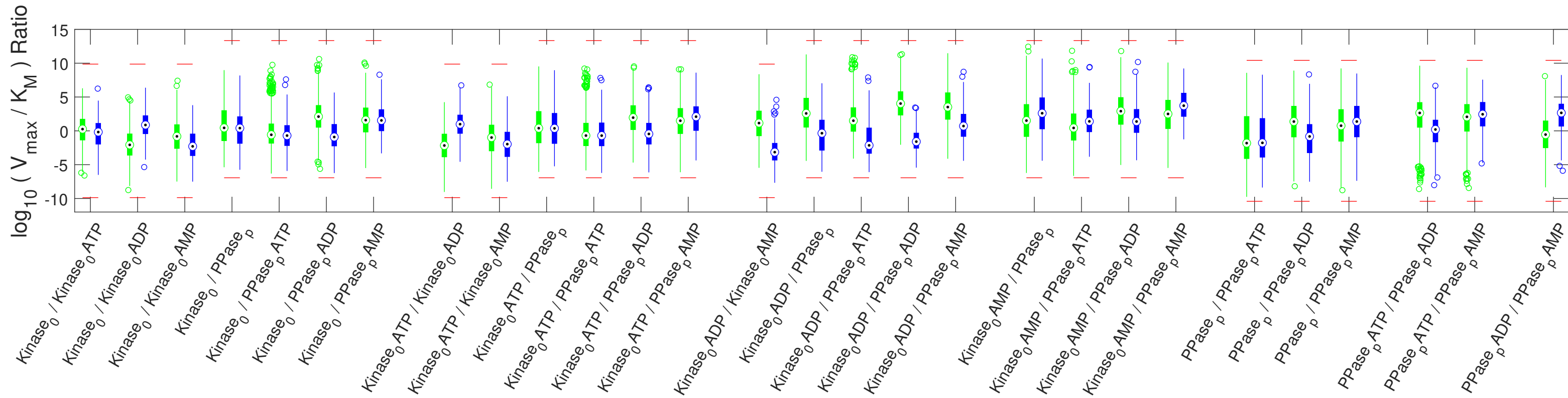

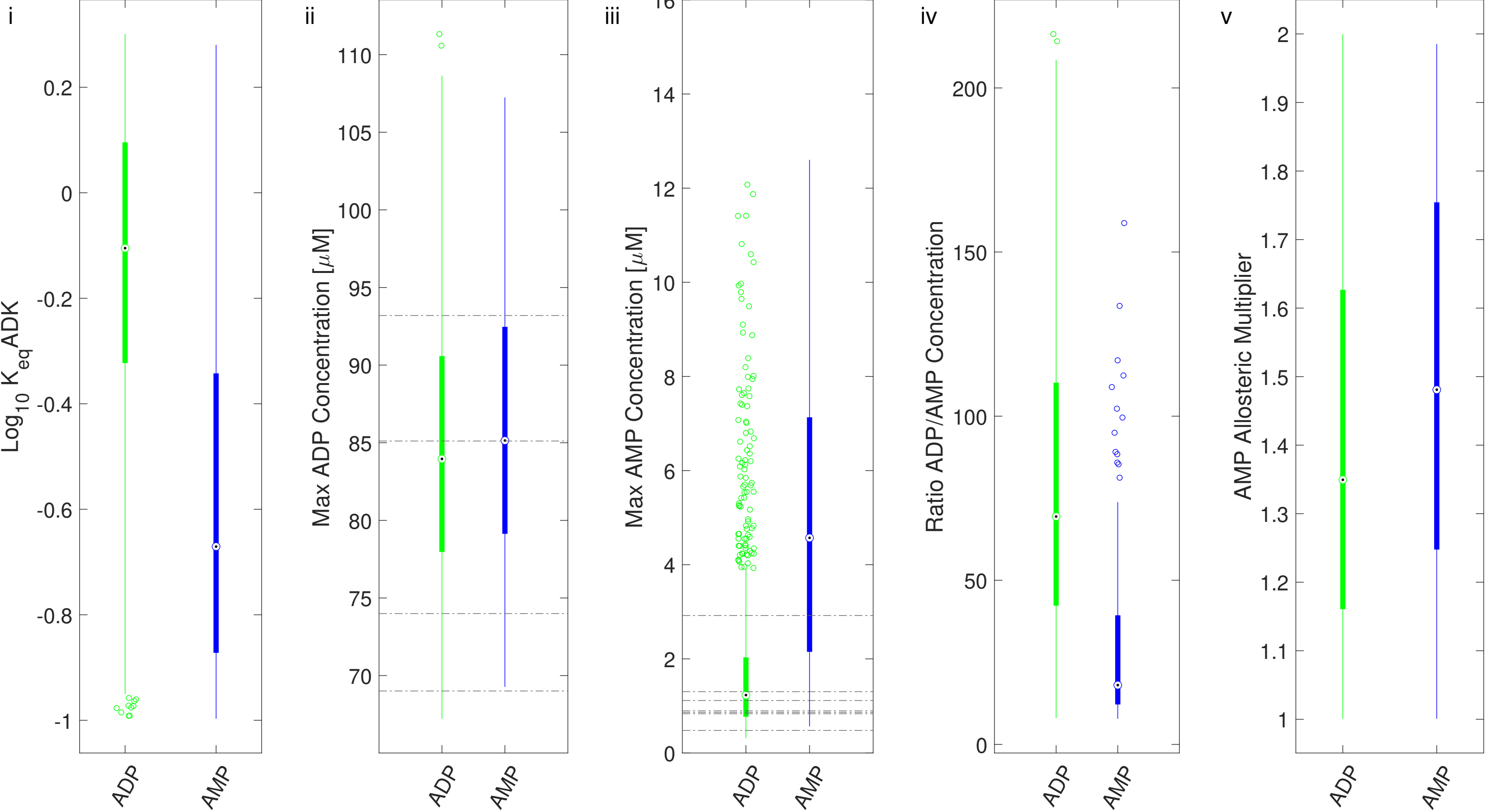

Supplement: S3 Fig — A. Effects of AXP-(p)-AMPK binding constants on AMP- versus ADP-dominant control of AMPK activity. i) Boxplots summarize the individual parameter values representing the forward (kf) and reverse (kr) rate constants. ii) Boxplots summarize the dissociation constants (KD) for each AXP-(p)-AMPK reaction, calculated as kr/kf from each simulation. iii) Boxplots summarize the ratios of all possible pairs of KD values. B. Effects of parameters determining the enhancement of AMPK phosphorylation on AMP- versus ADP-dominant control of AMPK activity in the α2β2γ3 KD MPSA. i) Boxplots summarize the Michaelis constants (KM) and maximum enzyme velocities (Vmax) for the AMPK kinase and phosphatase. ii) Boxplots summarize the catalytic efficiencies of the AMPK kinase and phosphatase, which was calculated as Vmax/KM for each simulation. iii) Boxplots summarize the compound ratios of kinase and phosphatase catalytic efficiencies. C. Effects of KeqADK, AMP and ADP concentrations, and allostery on AMP- versus ADP-dominant control of AMPK activity in the α2β2γ3 MPSA. Panels from left to right: i) Boxplots summarize the KeqADK values. ii) and iii) Boxplots summarize the maximum predicted concentrations of ADP (ii) and AMP (iii) during simulated exercise. The dashed-grey horizontal lines represent the reported maximum levels of ADP or AMP in moderate-intensity exercise studies. iv) Boxplots summarize the ratios of the maximum concentrations of ADP and AMP during simulated exercise. v) Boxplots summarize the magnitudes of allosteric activation of the AMP-p-AMPK complex. (PDF) [file pcbi.1008079.s006.pdf]

— ADP p-AMPK  
— AMP p-AMPK  
— Total AMPK

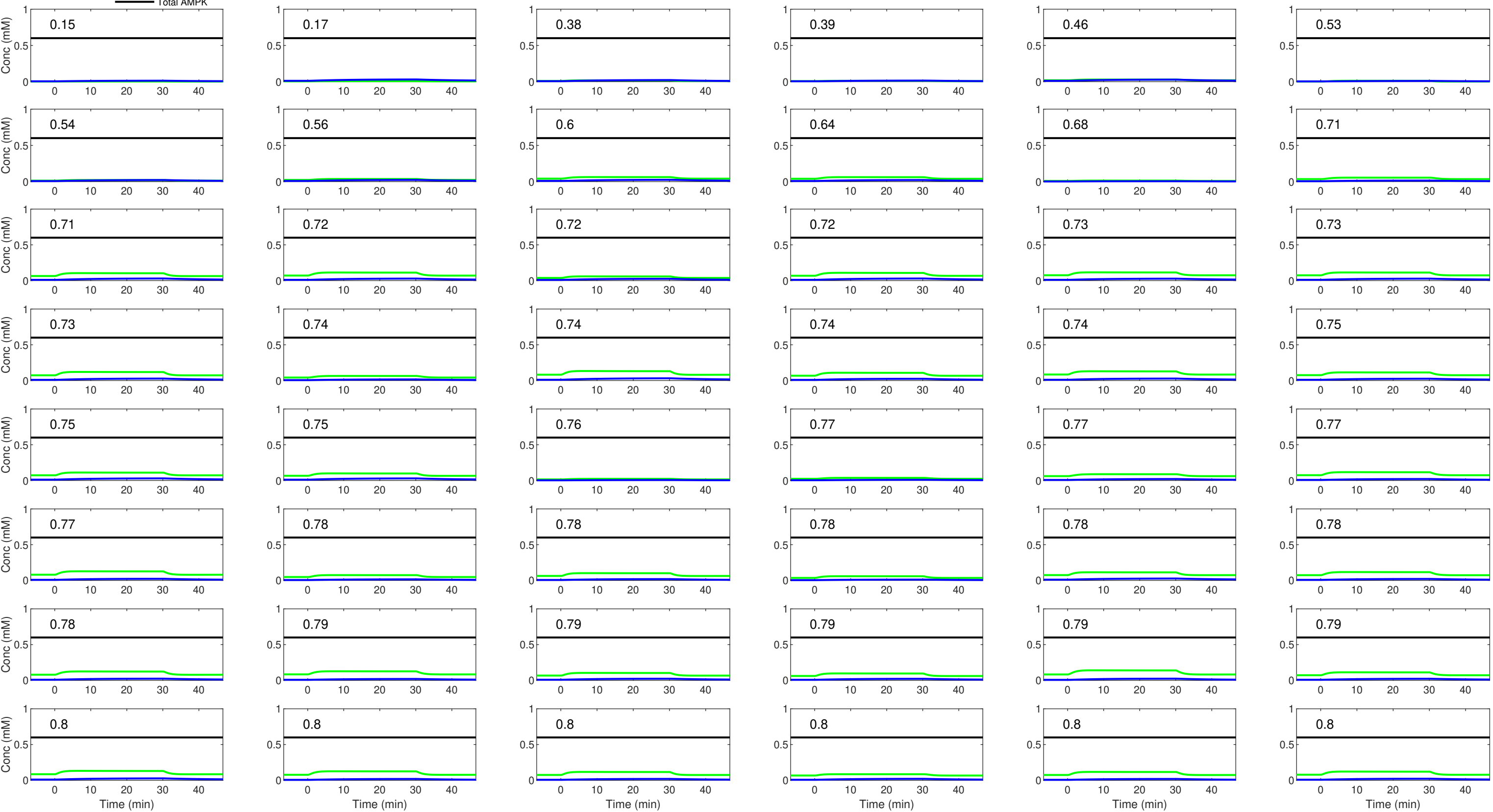

Supplement: S4 Fig — Model-predicted time courses of AMP-p-AMPK and ADP-p-AMPK in the 48 models remaining after the elimination process (see Fig 4 of the main text). The blue, green, and black lines represent the levels of AMP-p-AMPK, ADP-p-AMPK and total AMPK, respectively. The inset numbers represent the corresponding fraction of ADP control. (PDF) [file pcbi.1008079.s007.pdf]
