# Supplementary material for: ADP is the dominant controller of AMP-activated protein kinase activity dynamics in skeletal muscle during exercise
Source: PLoS Comput Biol. 2020 Jul 30;16(7):e1008079. doi: 10.1371/journal.pcbi.1008079 (PMC7433884; doi:10.1371/journal.pcbi.1008079)
Supplement: Attachment — Submitted filename: Coccimiglio_AMPK model_2020 vR1 review response.pdf [file pcbi.1008079.s008.pdf]

Response to reviewer comments

We thank each of the reviewers for their constructive critical comments that have helped to improve the manuscript.

Please note that all citations to line numbers in the revised manuscript are based on the manuscript file with tracked changes, and not the clean version.

Reviewer #1 comments:

The manuscript is well-written, is based on extensive and relevant literature, and employs robust validation of their model to provide confidence in their conclusion that ADP imparts the larger contribution to triggering AMPK activation. Limitations are readily acknowledged and discussed.

We thank the reviewer for his positive impressions of our work.

Why is consideration not given to AMP clearance mechanisms? e.g. AMP deaminase reaction, which influences the degree to which the AMP/ATP ratio increases during metabolic stress. Plaideau, C., et al (2012). doi: 10.1096/fj.11-198168. I note this is referred to in Fig 1 but not included in the model. Thus changes in [AMP] are likely to be lower than that predicted from the AK reaction alone. Of course, this will not alter the main conclusion of the study, however I consider this to be an important consideration for model refinement and one that is often over-looked by researchers in arguments supporting the AMP-centric hypothesis, or predicting AMP levels from measured ADP/ATP ratio. At the very least this should be included in the Introduction in line 96, and will likely improve accuracy of the model (e.g. predicted vs measured [AMP] fig 2B).

We concur with the reviewer's comment and have ongoing efforts to expand the model to include the AMP deaminase reaction. However, doing so requires that we also include a counterbalancing synthesis reaction to resupply the adenine nucleotide pool, as well as regulatory mechanisms for AMP deaminase to enable the revised model to fit data at rest and during exercise at different intensities. These modifications have been nontrivial to implement, and their inclusion would detrimentally increase the scope of the present study. Given that omitting this reaction does not significantly affect our conclusions, we are reserving this work for a future publication.

We revised the manuscript text to incorporate the reviewer's comment. He suggested mentioning the AMP deaminase reaction on line 96, but that paragraph is devoted to arguments supporting AMP as the dominant controller of AMPK, whereas the following paragraph is devoted to arguments supporting ADP as the controller. We therefore edited the latter paragraph to include the following sentence beginning on line 111: "The disparity in the intracellular concentrations of ADP and AMP is a manifestation of the adenylate kinase reaction operating near equilibrium and is further promoted by the AMP deaminase reaction, in which AMP is degraded into inosine monophosphate (IMP) and ammonia [1,2]. This reaction is particularly discernible during maximal-intensity sprint exercise, because intramuscular IMP levels increase from undetectable levels at rest to ~20 μmol·g dry mass^{-1} after exercise [3]."

An important component of model validity is selection of appropriate kinetic data. For a2b2g3, why did the authors select allostery data from Rajamohan et al ref 26 in preference to Ross et al ref 42 (Table S5)? The former provides data at sub-physiological ATP 20 uM using bacterial-expressed AMPK, whereas ref 42 employed a range of ATP concentrations up to 5 mM and mammalian cell expressed AMPK. It should be noted in the Introduction line 99 that g3 complexes are generally regarded to be poorly AMP-sensitive. Difference in fold activation between the two studies may be small (1.4 vs. 1.54), but will likely have a large influence on outputs.

We appreciate the reviewer's comment and acknowledge that some data are better than others. We explain our inclusion of the Rajamohan data as follows: Once we observed that ADP-p-AMPK complexes vastly outnumbered AMP-p-AMPK (Figures 2D, 3D, 3H, 3L), our next objective was to rigorously test the hypothesis that ADP was the dominant controller. A stringent way to test this hypothesis using modeling is to simulate models in a manner that includes the full scope of uncertainties for each parameter value. Accordingly, we deliberately sampled from the full ranges of reported parameter values, regardless of their experimental source, even if that meant including values that would lead to results that were unfavorable to the hypothesis. This approach led us to observe that ADP was dominant for most parameter sets, thus robustly supporting the hypothesis.

Regarding the reviewer's final point, this approach enabled us to learn that changing the allosteric activation of AMPK from 1.54 to 1.4-fold causes only a small decrease in the probability that the corresponding parameter set leads to strong AMP or ADP dominance (see Figure S3C, rightmost panel, which shows that many of the ADP-dominant simulations had AMP-allosteric-activation potencies of 1.5 or higher).

The following six comments made by the reviewer pertained to three successive paragraphs in the Introduction, such that we addressed them collectively. We incorporated all the comments by extensively editing the three paragraphs, which we have pasted below the sixth comment. The yellow highlights indicate the changes we made to address each of the specific comments.

line 84: AMPK has several known regulatory phosphosites, the authors need to be more specific here. Suggest changing to "ADP and AMP inhibit AMPK pThr-172 dephosphorylation and promote AMPK Thr-172 phosphorylation by LKB1".

Change made, please see below.

line 84 & 97. It should be mentioned that AMP and ADP stimulate Thr172 phosphorylation by CaMKKB (Oakhill et al 2010 PNAS doi/10.1073/pnas.1009705107 and ref 18) although I do not propose this element be incorporated into the modelling.

Change made, please see below.

line 85: include ref 26 (1st to show ADP protects against pT172 dephosphorylation).

References now included.

line 96-8: this sentence could be misinterpreted, AMP does not affect LKB1 activity directly, but instead makes AMPK T172 a better substrate for upstream kinases.

Change made, please see below.

line 105-8: consider changing to "…ADP-mediated net increase in pThr172…"to include both phos and dephos mechanisms.

Sentence replaced altogether in the rewrite of the paragraph.

lines 111 & 112: this concept was introduced in ref 24, not 18.

Change made, please see below.

<u>The edited paragraphs in the revised manuscript now read as follows (starting at Line 87)</u>:

> ADP and AMP inhibit phospho-AMPK Thr172 dephosphorylation [4,5] and promote AMPK Thr172 phosphorylation by LKB1 and CaMKKβ by enhancing AMPK's ability to serve as a substrate for these upstream kinases [2,6–8]. ATP inhibits AMPK phosphorylation by competing with ADP and AMP for binding to AMPK and by stabilizing it in a conformation that favors its dephosphorylation [9]. AMP allosterically activates AMPK catalytic activity by 1.5- to 13-fold, depending on the estimate, by binding to cystathionine-β-synthase (CBS) domains within the γ subunit [5,6,8]. How these factors interact to control overall AMPK activity is poorly understood.
>
> Two principal hypotheses have been proposed to explain AXP-mediated control of AMPK activity *in vivo*. One hypothesis posits that AMP (or its ratio with ATP) is the primary controller of AMPK [10,11]. Three lines of argument support this hypothesis. First, AMP is proposed to act as a more sensitive controller of AMPK activity than ADP for a given amount of ATP breakdown because the adenylate kinase reaction involves ATP and AMP interconverting into two ADP, such that the AMP:ATP ratio would vary as the square of the ADP:ATP ratio [12]. Second, AMP enhances the phosphorylation of AMPK by LKB1 and by CaMKKβ by up to four fold [6–8], whereas ADP enhances this phosphorylation by only ~1.5-fold [2,6]. Third, only AMP allosterically activates AMPK [5,6,8]. Overall, AMP is more potent on a per-molar basis than ADP in activating AMPK [8,11], which ultimately reflects a higher *per-molecule activation potency*, i.e., the degree of activation per AMPK molecule upon the binding of activator.

> An alternative hypothesis is that ADP is the primary controller of AMPK activity, which is based on two main arguments. First, the concentration of free ADP is orders of magnitude higher than that of free AMP (50-200 μM versus 0.5-5 μM) [2,4,9,13,14]. The disparity in the intracellular concentrations of ADP and AMP is a manifestation of the adenylate kinase reaction operating near equilibrium and is further promoted by the AMP deaminase reaction, in which AMP is degraded into inosine monophosphate (IMP) and ammonia [1,2]. This reaction is particularly impactful during maximal-intensity sprint exercise, because intramuscular IMP levels increase from undetectable levels at rest to ~20 μmol·g dry mass^{-1} after exercise [3]. Second, ADP and AMP both bind to the AMPK γ-subunit nucleotide-binding site 3 with roughly similar affinities [4]. Data from human exercise studies show that the concentration of ADP exceeds its dissociation constant (K_D) of binding to site 3 on the γ domain, whereas the concentration of AMP does not, thus implying that ADP outcompetes AMP for binding to that site [13]. In accordance, ADP levels and AMPK activities increase in parallel as a function of exercise intensity [13]. Together, the comparable affinities of binding and the higher ADP concentration may enable it to serve as the primary controller of AMPK despite its lesser per-molecule activation potency compared to AMP.

line 431…."164 simulations….." should this read "plausible"?

We reread the paragraph and noticed an error that may have led to confusion and prompted the reviewer's comment. Specifically, on line 429 of the original manuscript, we stated "164 simulations…" and this should have read "201 of these simulations". On line 431, we stated "Of the 164 remaining models…", which is correct and should be more understandable now that the prior error was corrected, especially given that the numbers now align with those listed in Figure 4A.

The corrected passage can be found on line 476 of the revised manuscript.

line 501: for clarity suggest changing to…"The 10-2 mM dose of C991 elicited lower phospho-AMPK levels but higher AMPK activity, when compared to AICAR and moderate exercise (Fig. 5B and 5C)". The authors could mention this is consistent with the view that synthetic AMPK agonists elicit robust AMPK signalling without affecting Thr172 phosphorylation (Scott et al 2015, http://dx.doi.org/10.1016/j.chembiol.2014.03.006).

Change made, see line 519 of revised manuscript.

ref 26 and 42 are identical.

Corrected.

Reviewer #2 comments:

This is a well-written manuscript and the results may have significance for understanding what cellular signals drive muscle plasticity. In addition, there is significance for pharmaceuticals and dietary supplements, "exercise mimetics" that include small molecule activators of AMPK.

We thank the reviewer for their positive impressions of our work.

My biggest concern about this manuscript is what the authors consider to be the physiological range of free, cytoplasmic [ADP]. This may have a major impact on the authors' conclusion. The concentration range of AMP specified on page 5, line 6 from bottom, seems off (specifically too high) at the lower end. For example, see Kushmerick et al., 1992, Mammalian skeletal muscle fibers distinguished by contents of phosphocreatine, ATP, and Pi. Proc. Natl. Acad. Sci. USA 89:7521-7525. There is additional relevant ref data in related papers from the Kushmerick lab, e.g., Chase and Kushmerick, 1995, Effect of physiological ADP levels on contraction of single skinned fibers from rabbit fast and slow muscles. Am. J. Physiol. 268:C480-C489. The authors' argument that "ADP is the dominant controller" relies in part on their higher concentrations of ADP relative to AMP (in combination with affinities). My reading of the 31P NMR literature on muscle suggests that the range of ADP indicated by the authors would only be found during heavy exercise or hypoxia. The authors therefore need to clarify what happens at lower ADP levels and whether their conclusion would change; perhaps in a 3D plot define the concentration ranges over which ADP or AMP would dominate.

Indeed, the concentration estimates of the adenine nucleotides are critical to our conclusions. We address the reviewer's comments by stating the following three points:

1) The concentrations of ADP and AMP ***relative to each other*** are the critical factor driving system behavior, rather than the absolute values of either alone. These concentrations are positively correlated due to the adenylate kinase reaction, such that models featuring lower ADP concentrations tend to feature correspondingly lower AMP concentrations.
2) Our model reproduces measured ADP concentrations in skeletal muscle during rest and exercise.
3) Slight discrepancies are observed between NMR and biochemically based measurements of ADP, with the former tending to feature lower values.

The details underlying these points are as follows:

1) The global sensitivity analyses demonstrated that varying the parameters caused the maximum ADP and AMP concentrations to vary, and both ADP and AMP dominant cases were observed over a range of maximum absolute concentrations for both (Figures S1C, S2C, S3C; second and third panels of each). Dominance was determined more by their ***relative concentrations***, which is evident by the clear shift of the boxplots associated with ADP and AMP dominant cases for the *Ratio of ADP/AMP Concentration* in Figures S2C and S3C (fourth panel of each)[1].

   This result accords with theory: ADP and AMP concentrations are connected through the adenylate kinase reaction. Lower estimates of ADP would coincide with lower estimates of AMP, with the relative difference dependent on the K_{eqADK} value. The latter value was plotted in the leftmost panels of Figures S1C, S2C, and S3C. It is evident that the shifts in the boxplots for K_{eqADK} and for *Ratio ADP/AMP Concentration* are similar.

   To demonstrate this effect using our model, we adjusted the model to reduce the resting ADP concentration to 22.5 μM, and resimulated it according to the "Nielsen Sedentary" exercise protocol. ADP is still clearly dominant in this case (compare ADP-p-AMPK with AMP-p-AMPK in panel D of the Figure below).

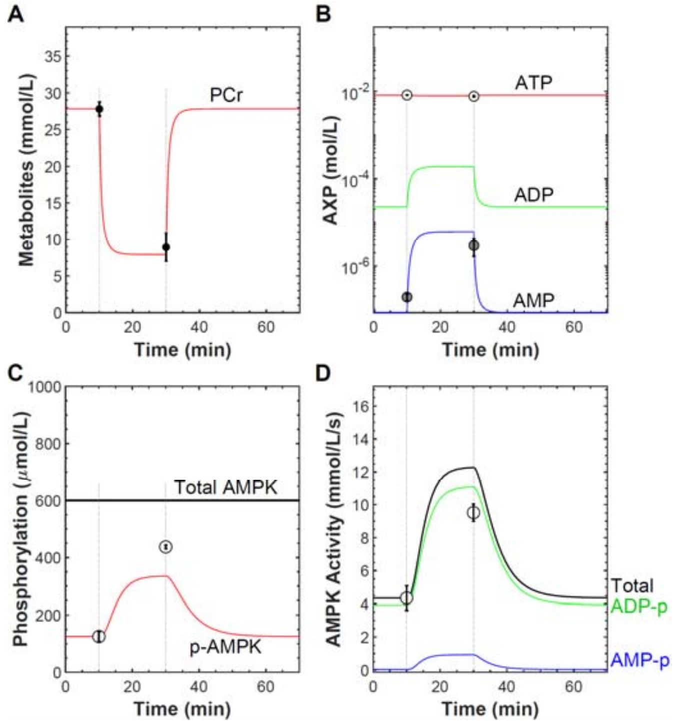

Figure. Resimulation of the Neilsen Sedentary protocol with lower ADP levels. In this simulation, the pH was reduced from 7.2 to 7, thus causing the K_{eqCK} to increase from ~112 to 177, which resulted in the lower resting ADP concentration of 22.5 μM (panel B). k_{stim} and k_{rest} were adjusted to ensure the fit of the PCr data. ADP-p-AMPK levels still far exceeded AMP-p-AMPK values during simulated exercise (panel D).

[1] In Figure S1C, which corresponds to the unconstrained MPSA, changes in the AXP-(p)-AMPK K_D were the primary factor determining AXP dominance, such that the boxplots related to concentrations are similar for ADP and AMP-dominant cases.

2) The calibrated model demonstrates the satisfactory fit of the model to ADP measurements presented in Stephens et al. [15] (Figure 2B, green line) at both rest (49 µM vs. 57 µM for the model and data, respectively) and during exercise (91 µM vs. 93 µM for the model and data, respectively).

3) We tabulated ADP concentration estimates in human muscle at rest and exercise from several studies. Free ADP is calculated from measurements of the other reactants in the creatine kinase reaction. These measurements were made either biochemically (from muscle biopsies) or using nuclear magnetic resonance (NMR) techniques.

Reference	Species	Condition	Measurement method	Free [ADP] (µM)*
Stephens 2002 [15]	Human	Rest	biochemical	57
Chen 2003 [16]	Human	Rest	biochemical	27
McConell 2005 [17]	Human	Rest, low CHO diet	biochemical	50
	Human	Rest, high CHO diet	biochemical	42
Hellsten 1999 [18]	Human	Rest	biochemical	18
Veech 1979 [19]	Rat muscle	Rest	biochemical	37
Malucelli 2011 [20]	Human	Rest	NMR	62
Vicini 2000 [21]	Human	Rest	NMR	16
Kushmerick 1998 [22]	Mammalian	Rest	Assumed (model)	10
Cited data in Chase 1995 [23]	Vertebrate (frog, cat, rat, mouse, human)	Rest	NMR	1-10
		Exercise	NMR	50-300

* Biochemically obtained estimates were calculating by converting the reported units (µmol·kg dry mass^{-1}) into µM using the conversion factor 3.13 L/kg [24].

The data suggest that estimates of free [ADP] vary somewhat between studies but are consistently in the low micromolar range (1-60 µM). Measurements obtained from biochemical measurements in muscle biopsies tend to be higher than those obtained via NMR.

In our case, we sought to enhance our ability to compare the model predictions to data in an internally consistent manner, such that we selected calibration and validation data from studies in which both metabolites and AMPK signaling were measured ***from the same muscle biopsies***.

Altogether, we stand by the [ADP] estimates used in our study and we contend that our conclusions are robust to variations in measured [ADP].

In the concluding line of Abstract, clarify "ADP is the dominant controller of AMPK activity dynamics in skeletal muscle during exercise by virtue of its higher concentration." Specifically, clarify "higher concentration" relative to what (AMP) as stated more clearly in the author summary.

Change made as requested.

In the Author Summary: "How the adenine nucleotides interact to control AMPK activity is poorly understood." Clarify or eliminate because there are plenty of 3D structures and in vitro biochemistry.

We changed the sentence to read "How the adenine nucleotides interact to control AMPK signaling dynamics is incompletely understood."

Reviewer #3 comments:

This paper deals with an important issue, and uses a computational approach to draw some interesting conclusions.

We appreciate the reviewer's positive impressions of our work.

I'd like to see a little more detail about the bioenergetic module, which is basically Vicini plus Lambeth (to use a lazy shorthand). Lambeth models glycogenolysis to lactate in a closed system at constant pH (assuming a fixed glycogen phosphorylase a/(a+b) ratio), Vicini models oxidative phosphorylation, and both include the creatine kinase system. The present paper I think assumes no lactate production (i.e. pyruvate is fully oxidised) and although pH-dependence of various model parameters is (rightly) included explicitly, the actual pH is fixed in the model.

- It would be useful to have this explicitly stated in the Supplement, along with the reasons for the simplifications.
- 'The module includes…' section in the Supplement should presumably contain glycolysis.

The reviewer's comments refer primarily to the "Bioenergetic module" passage of the "Model construction: Topology" subsection of the Supplementary Methods. In rereading that passage, we agree with the reviewer that our description of how we incorporated elements of the Lambeth model was unclear. We have edited the original paragraph and added a second paragraph to make explicit what was included and what was omitted from the model:

> *The bioenergetic module*. The bioenergetic module is based on the previously validated model of Vicini et al. [21], and incorporates the adenylate kinase (AK) reaction from the model of Lambeth et al. [25]. This module explicitly models ATP hydrolysis (Fig. 1 and Table S2, reaction r1) and the ATP supply processes of oxidative phosphorylation (Fig. 1 and Table S2, reaction r2), the creatine kinase reaction (CK; Fig. 1 and Table S2, reactions r3 and r4), and the AK reaction (Fig. 1 and Table S2, reaction r5). The rate equations and pre-calibration initial conditions for these reactions were obtained from the source models [21,25].
>
> The bioenergetic module features the following simplifications to foster parsimony. First, we assumed a constant total adenine nucleotide pool: we omitted the AMP degradation reaction to inosine monophosphate (IMP) and ammonia [1] and the various counterbalancing synthesis reactions. Second, we did not include glycolysis and lactate formation, which supply ATP and NADH. We also omitted modeling dynamic changes in pH but instead set pH at a constant level. These simplifications were justified on theoretical grounds because the primary purpose of the bioenergetic module was to satisfactorily replicate AXP dynamics, which served as inputs into the AMPK regulatory module, rather than faithfully replicate all aspects of muscle metabolism. We empirically validated these assumptions by comparing the model predictions to data and by performing sensitivity analyses, as described in the Results.

- To be clear, this approach seems reasonable to me, and unlikely to affect the novel results which are the point of the paper. The one possible exception is pH: you do discuss this (p. 22) but I'm not sure why you conclude '.. thus reinforcing our conclusions' (line 635).

We are pleased that the reviewer concurs with our approach. We concluded that the pH assumptions "reinforced" our conclusions indirectly because lowered pH favors ADP formation as opposed to AMP, as explained in that paragraph. In retrospect, we now see that the statement could be misinterpreted. Accordingly, we have revised it as follows (see line 652 of revised manuscript):

> Collectively, these effects would reduce AMP concentration proportionally more than that of ADP, which would favor ADP-dominant control of AMPK activity. Accordingly, the dynamic pH changes observed in working skeletal muscle are unlikely to affect our conclusions.

A more general comment on the physiological context. I agree that 'Enhanced understanding of AMPK activation would inform both exercise training biology and AMPK pharmacology'. I'm struck by the difference in time-scale between three things: (i) AMPK activation by ADP and AMP (which go up and down, roughly together, in potentially complicated patterns as the myocyte happens to start, stop or change the intensity of contractions; and much of the time are around resting levels), (ii) the downstream consequences, dependent on complex series of gene activations, and (c) AMPK activation by potentially therapeutic pharmacological agents.

The reviewer poses an excellent question, and it is one that is central to the senior author's research program!

We are also curious about the apparent "separation of timescales" observed for i) and ii); indeed, signaling operates on a relatively fast timescale (minutes to hours), transcription operates on a timescale of hours, and protein synthesis on a timescale of hours to days. The interplay of these processes in response to exercise training was clarified in an elegant study by Perry et al. [26]. The progressively slower kinetics of these processes are such that repeated stimuli (individual exercise training sessions) performed at sufficient frequency enable the gradual but relatively stable accumulation of the final product of the pathway, i.e., fitness-promoting proteins and cellular structures and organelles. We have ongoing theoretical and experimental work to characterize these dynamics and demonstrate this principle.

It should also be mentioned that AMPK stimuli beyond the adenine nucleotides may exist and continue to operate after the cessation of exercise, such that the apparent separation of timescales may not be as stark. These stimuli, such as glycogen depletion [27], increased body temperature [28], autocrine/paracrine/endocrine IL-6 signaling [29], could promote AMPK signaling during the recovery period from exercise. However, the data supporting these possibilities as major contributors to AMPK signaling are not particularly strong, such that these effects are likely small relative to those of the adenine nucleotides.

Presumably what relates (i) and (ii) is just the averaging/integrating effect of multiple processes. Does this model have anything to say about that?

This study does not make any suppositions for how AMPK signaling dynamics are translated into adaptations. However, we have ongoing computational and experimental work to determine the signaling properties that predict adaptation. It is reasonable to hypothesize that the *area under the curve* of AMPK activity is the primary factor [30], but if downstream processes vary with higher-order kinetics as a function of signaling, then the possibility exists that other features of the signaling dynamic might be more predictive of their kinetics and eventual adaptation [see ref 31 for a review of how signaling dynamics encode information].

What is the best way of thinking of the relationship of the pharmacology of (iii) to that physiology?

AMPK physiology and pharmacology are analogous. Exercise acts like a drug [32], and drugs are being developed to mimic exercise [33]. The link between the two is pharmacokinetics: in the case of AMPK, exercise elicits a time course of the activator molecules (ADP, and to a lesser extent AMP), whereas administration of pharmacological activators of AMPK such as AICAR will lead to a time-dependent concentration profile in blood that perfuses the tissues and exposes the cells to the drug.

Pharmacokinetic studies of AICAR administered in human participants reveal a half-life of 1-2 hours [34], which corresponds approximately to the duration of prolonged endurance training workouts that well-trained athletes commonly undertake. Equating intensity with concentration of drug is challenging without more data on both. Repeated AICAR infusion has been attempted in humans to address metabolic diseases such as diabetes but with mixed results [35,36].

In any case, our model could be combined with pharmacokinetic models of molecules that target AMPK to predict the effect on signaling. Such physiologically-based pharmacokinetic-pharmacodynamic (PB-PKPD) models are the central tools of the nascent field of quantitative systems pharmacology [37].

Fig 6 simulates direct activation of AMPK by infused activators (alone and together), but how would that interact with the exercise response?

The interplay would be complex and would depend on the mechanism of action of the drug. Some activators like ZMP (the metabolite of AICAR) bind to adenine nucleotide sites on AMPK, such that they compete for binding with the endogenous activators. Other activators, such as compound 991, bind elsewhere on AMPK, such that additivity, antagonism, or synergism are all possibilities. Our model would have to be extended to include all these possibilities.

Refs 35 and 39 are the same

Corrected.

Something about the phrasing of 'Reduced pH provides a driving force away from ADP and towards ATP in the creatine kinase reaction' doesn't seem quite appropriate to an equilibrium. If Cr/PCr is unchanged, yes, a lower pH implies a lower [ADP], but embedded in the whole metabolic regulatory network that condition can't be assumed.

We acknowledge the reviewer's point and agree that changes in the concentrations of individual reactants cannot be straightforwardly predicted for reactions embedded in interconnected dynamic systems. There are, however, studies that support our contention [e.g., see ref 38]. We therefore replaced the sentence with the one that follows to state our point more specifically and to incorporate the reviewer's comment (line 646, revised manuscript):

> Because this reaction operates close to equilibrium in vivo [19], decreased intracellular pH is thought to contribute to decreased free ADP concentration [38,39].

References

1. Sahlin K, Broberg S. Adenine nucleotide depletion in human muscle during exercise: causality and significance of AMP deamination. Int J Sports Med. {\copyright} Georg Thieme Verlag Stuttgart·New York; 1990;11: S62--S67.
2. Oakhill JS, Steel R, Chen Z-P, Scott JW, Ling N, Tam S, et al. AMPK is a direct adenylate charge-regulated protein kinase. Science. 2011;332: 1433–5. doi:10.1126/science.1200094
3. Karatzaferi C, De Haan A, Ferguson R, Van Mechelen W, Sargeant A. Phosphocreatine and ATP content in human single muscle fibres before and after maximum dynamic exercise. Pflügers Arch. Springer; 2001;442: 467–474.
4. Xiao B, Sanders MJ, Underwood E, Heath R, Mayer F V, Carmena D, et al. Structure of mammalian AMPK and its regulation by ADP. Nature. 2011;472: 230–3. doi:10.1038/nature09932
5. Suter M, Riek U, Tuerk R, Schlattner U, Wallimann T, Neumann D. Dissecting the role of 5'-AMP for allosteric stimulation, activation, and deactivation of AMP-activated protein kinase. J Biol Chem. 2006;281: 32207–16. doi:10.1074/jbc.M606357200
6. Ross FA, Jensen TE, Hardie DG. Differential regulation by AMP and ADP of AMPK complexes containing different γ subunit isoforms. Biochem J. 2016;473: 189–99. doi:10.1042/BJ20150910
7. Oakhill JS, Chen Z-P, Scott JW, Steel R, Castelli LA, Ling N, et al. β-Subunit myristoylation is the gatekeeper for initiating metabolic stress sensing by AMP-activated protein kinase (AMPK). Proc Natl Acad Sci U S A. 2010;107: 19237–41. doi:10.1073/pnas.1009705107
8. Gowans GJ, Hawley SA, Ross FA, Hardie DG. AMP is a true physiological regulator of AMP-activated protein kinase by both allosteric activation and enhancing net phosphorylation. Cell Metab. 2013;18: 556–66. doi:10.1016/j.cmet.2013.08.019
9. Carling D, Thornton C, Woods A, Sanders MJ. AMP-activated protein kinase: new regulation, new roles? Biochem J. 2012;445: 11–27. doi:10.1042/BJ20120546
10. Winder WW. Energy sensing and signaling by AMP-activated protein kinase in skeletal muscle. J Appl Physiol. 2001;91: 1017–1028.
11. Gowans GJ, Hardie DG. AMPK: a cellular energy sensor primarily regulated by AMP. Biochem Soc Trans. 2014;42: 71–5. doi:10.1042/BST20130244
12. Hardie DG, Hawley SA. AMP-activated protein kinase: the energy charge hypothesis revisited. Bioessays. 2001;23: 1112–1119. doi:10.1002/bies.10009 [pii] 10.1002/bies.10009
13. Oakhill JS, Scott JW, Kemp BE. AMPK functions as an adenylate charge-regulated protein kinase. Trends Endocrinol Metab. Elsevier Ltd; 2012;23: 125–132. doi:10.1016/j.tem.2011.12.006
14. Hardie DG, Carling D, Gamblin SJ. AMP-activated protein kinase: also regulated by ADP? Trends Biochem Sci. 2011/07/26. 2011;36: 470–477. doi:S0968-0004(11)00095-8 [pii]10.1016/j.tibs.2011.06.004
15. Stephens TJ, Chen Z-P, Canny BJ, Michell BJ, Kemp BE, McConell GK. Progressive increase in human skeletal muscle AMPKalpha2 activity and ACC phosphorylation during exercise. Am J Physiol Endocrinol Metab. 2002;282: E688–E694. doi:10.1152/ajpendo.00101.2001

16. Chen ZP, Stephens TJ, Murthy S, Canny BJ, Hargreaves M, Witters LA, et al. Effect of exercise intensity on skeletal muscle AMPK signaling in humans. Diabetes. 2003;52: 2205–2212. doi:10.2337/diabetes.52.9.2205

17. McConell GK, Lee-Young RS, Chen Z-P, Stepto NK, Huynh NN, Stephens TJ, et al. Short-term exercise training in humans reduces AMPK signalling during prolonged exercise independent of muscle glycogen. J Physiol. 2005;568: 665–676. doi:10.1113/jphysiol.2005.089839

18. Hellsten Y, Richter EA, Kiens B, Bangsbo J. AMP deamination and purine exchange in human skeletal muscle during and after intense exercise. J Physiol. Wiley Online Library; 1999;520: 909–920.

19. Veech RL, Lawson JW, Cornell NW, Krebs H a. Cytosolic phosphorylation potential. J Biol Chem. 1979;254: 6538–6547.

20. Malucelli E, Iotti S, Manners DN, Testa C, Martinuzzi A, Barbiroli B, et al. The role of pH on the thermodynamics and kinetics of muscle biochemistry: An in vivo study by 31P-MRS in patients with myo-phosphorylase deficiency. Biochim Biophys Acta (BBA)-Bioenergetics. Elsevier; 2011;1807: 1244–1249.

21. Vicini P, Kushmerick MJ. Cellular energetics analysis by a mathematical model of energy balance: estimation of parameters in human skeletal muscle. Am J Physiol Cell Physiol. 2000;279: C213-24. Available: http://www.ncbi.nlm.nih.gov/pubmed/10898733

22. Kushmerick MJ. Energy balance in muscle activity: simulations of ATPase coupled to oxidative phosphorylation and to creatine kinase. Comp Biochem Physiol B Biochem Mol Biol. 1998/10/27. 1998;120: 109–123. Available: http://www.ncbi.nlm.nih.gov/pubmed/9787781

23. Chase PB, Kushmerick MJ. Effect of physiological ADP concentrations on contraction of single skinned fibers from rabbit fast and slow muscles. Am J Physiol. 1995/02/01. 1995;268: C480-9. Available: http://www.ncbi.nlm.nih.gov/pubmed/7864087

24. Sjogaard G, Saltin B. Extra- and intracellular water spaces in muscles of man at rest and with dynamic exercise. Am J Physiol. 1982;243: R271-80.

25. Lambeth MJ, Kushmerick MJ. A computational model for glycogenolysis in skeletal muscle. Ann Biomed Eng. 2002;30: 808–827. doi:10.1114/1.1492813

26. Perry CGR, Lally J, Holloway GP, Heigenhauser GJF, Bonen A, Spriet LL. Repeated transient mRNA bursts precede increases in transcriptional and mitochondrial proteins during training in human skeletal muscle. J Physiol. 2010;588: 4795–4810. doi:10.1113/jphysiol.2010.199448

27. Wojtaszewski JFP, MacDonald C, Nielsen JN, Hellsten Y, Hardie DG, Kemp BE, et al. Regulation of 5′ AMP-activated protein kinase activity and substrate utilization in exercising human skeletal muscle. Am J Physiol Metab. American Physiological Society Bethesda, MD; 2003;284: E813--E822.

28. Liu CT, Brooks GA. Mild heat stress induces mitochondrial biogenesis in C2C12 myotubes. J Appl Physiol. 2011/11/05. 2012;112: 354–361. doi:japplphysiol.00989.2011 [pii]10.1152/japplphysiol.00989.2011

29. Ruderman NB, Keller C, Richard A-M, Saha AK, Luo Z, Xiang X, et al. Interleukin-6 regulation of AMP-activated protein kinase: potential role in the systemic response to exercise and prevention of the metabolic syndrome. Diabetes. Am Diabetes Assoc; 2006;55: S48--S54.

30. Schneider A, Klingmüller U, Schilling M. Short-term information processing, long-term responses: Insights by mathematical modeling of signal transduction: early activation dynamics of key signaling mediators can be predictive for cell fate decisions. Bioessays. Wiley Online Library; 2012;34: 542–550.

31. Purvis JE, Lahav G. Encoding and decoding cellular information through signaling dynamics. Cell. Elsevier; 2013;152: 945–956.

32. Vina J, Sanchis-Gomar F, Martinez-Bello V, Gomez-Cabrera M. Exercise acts as a drug; the pharmacological benefits of exercise. Br J Pharmacol. 2012;167: 1–12. doi:10.1111/j.1476-5381.2012.01970.x

33. Fan W, Evans RM. Exercise Mimetics: Impact on Health and Performance. Cell Metab. Elsevier; 2016;

34. Dixon R, Gourzis J, McDermott D, Fujitaki J, Dewland P, Gruber H. AICA-riboside: safety, tolerance, and pharmacokinetics of a novel adenosine-regulating agent. J Clin Pharmacol. Wiley Online Library; 1991;31: 342–347.

35. Bosselaar M, Smits P, van Loon LJC, Tack CJ. Intravenous AICAR during hyperinsulinemia induces systemic hemodynamic changes but has no local metabolic effect. J Clin Pharmacol. Wiley Online Library; 2011;51: 1449–1458.

36. Babraj JA, Mustard K, Sutherland C, Towler MC, Chen S, Smith K, et al. Blunting of AICAR-induced human skeletal muscle glucose uptake in type 2 diabetes is dependent on age rather than diabetic status. Am J Physiol Metab. American Physiological Society; 2009;296: E1042--E1048.

37. Piñero J, Furlong LI, Sanz F. In silico models in drug development: where we are. Curr Opin Pharmacol. Elsevier; 2018;42: 111–121.

38. Arnold DL, Matthews PM, Radda GK. Metabolic recovery after exercise and the assessment of mitochondrial function in vivo in human skeletal muscle by means of 31P NMR. Magn Reson Med. Wiley Online Library; 1984;1: 307–315.

39. Golding EM, Teague WE, Dobson GP. Adjustment of K'to varying pH and pMg for the creatine kinase, adenylate kinase and ATP hydrolysis equilibria permitting quantitative bioenergetic assessment. J Exp Biol. The Company of Biologists Ltd; 1995;198: 1775–1782.
