# Supplementary material for: ADP is the dominant controller of AMP-activated protein kinase activity dynamics in skeletal muscle during exercise
Source: PLoS Comput Biol. 2020 Jul 30;16(7):e1008079. doi: 10.1371/journal.pcbi.1008079 (PMC7433884; doi:10.1371/journal.pcbi.1008079)
Supplement: Attachment — Submitted filename: Coccimiglio_AMPK model_2020 vR2 review response.pdf [file pcbi.1008079.s009.pdf]

Response to reviewer comments

We thank again each of the reviewers for their constructive critical comments that have helped to improve the manuscript. We are pleased that our previous response allayed most of their concerns.

Reviewer #1 comments:

The authors have addressed all my concerns except one, which may have been missed: It should be noted in the Introduction line 99 that skeletal muscle gamma3 complexes have been shown to be poorly AMP-sensitive (in terms of allosteric activation) in studies that directly compare all 3 gamma isoforms eg Ross et al Biochem. J. 473, 189–199 (2016) and Langendorf et al Nat. Commun 7, 10912 (2016).

We acknowledge our oversight and have modified the text on line 102 as follows:

> Third, only AMP allosterically activates AMPK [1–3], the degree to which depends on the γ-subunit isoform within the AMPK complex, with γ2-containing complexes being strongly activated and γ3-containing complexes exhibiting little to no activation [1,4].

Reviewer #2 comments:

In the previous review, I stated that "My biggest concern about this manuscript is what the authors consider to be the physiological range of free, cytoplasmic [ADP]." In their responses, the authors state that "the concentration estimates of the adenine nucleotides are critical to our conclusions" and then state that it is the "concentrations of ADP and AMP relative to each other are the critical driving factor". The authors then present a simulation with resting ADP = 22.5 uM while their summary table of ADP estimates suggests that resting ADP may be significantly lower than 22.5 uM when we focus on the 31P NMR results.

I agree completely that the ratio of ATP/ADP is important for some aspects of metabolism, but the absolute concentrations can also be significant. I don't understand why they didn't perform the simulations at lower resting ADP (e.g., 1 and 10 uM) to demonstrate that their conclusions do not change, if that is the case.

We empathize with the reviewer's puzzlement and we should have better explained our approach in our original reply. We did not perform simulations at lower ADP concentrations because ADP concentration is not an independent variable that we can just set to any value; rather, it emerges from the model parameter values. To drive ADP concentration downwards, we have to modify parameter values such as the K_{eqCK} and k_{stim} and k_{rest} away from their nominal (and realistic) values, and excessively varying bioenergetic parameters can cause instability in the model. We felt that the previous simulation was a reasonable compromise given that we relied mainly on biopsy-based biochemical measurements for the data used for comparison, for which 22.5 µM was on the low end of the reported resting values.

I hope the authors would agree that good modeling practice is to work not only with parameters that are close to physiological, but in addition, to bracket key values with the range of physiological estimates. Beyond stating "we stand by the [ADP] estimates used in our study and we contend that our conclusions are robust to variations in measured [ADP]," the additional simulations at lower (but according to the 31P NMR data, still physiological) should only strengthen the authors' conclusions in the eyes of future readers if nothing changes qualitatively.

We agree in principle with the reviewer's statement; indeed, we designed the MPSA analyses to bracket physiologically plausible values for the parameters. However, in practice, this approach can lead to spurious results because some combinations of parameter values can lead to model instability, such that care is needed in defining the boundaries.

We generated two additional simulations of the "Nielsen Sedentary" exercise protocol, one in which resting ADP was ~10 µM and the other ~1 µM. To generate the 10 µM simulation, we adjusted K_{eqCK} from its nominal value of 111 to 396. During simulated exercise, ADP-p-AMPK levels exceeded those of AMP-p-AMPK by approximately 30-fold (Figure 1). To generate the 1 µM simulation, we adjusted the K_{eqCK} to 3,925, resulting in an even greater disparity between ADP-p-AMPK and AMP-p-AMPK (Figure 2, panel D), with simulated resting AMP

concentrations now in sub-nanomolar amounts (Figure 2, panel B). However, it is evident that the altered parameter values appear to have led to unstable model behaviors (Figure 2).

Between the calibration and validation procedures in the paper and in the response to reviews, we have now shown ADP dominance during simulated exercise across four starting concentrations of ADP (57 µM, 22 µM, 10 µM, and 1 µM). We hope that our explanation and new simulations satisfy the reviewer's concerns.

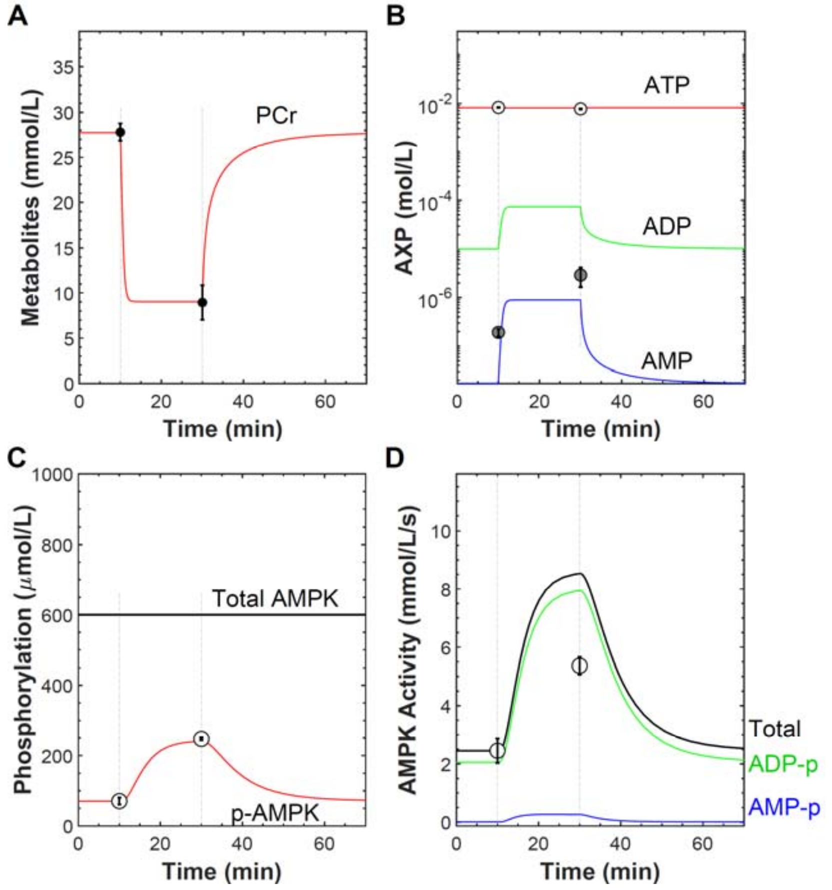

Figure 1. Simulation of the Nielsen Sedentary protocol with lower ADP levels (resting = ~10 µM, i.e., 10^{-5} M; panel B). In this simulation, the K_{eqCK} was increased to 396 from its nominal value of 111. k_{stim} and k_{rest} were then adjusted to ensure the fit of the PCr data. ADP-p-AMPK levels (green line) were higher relative to those of AMP-p-AMPK (blue line) during simulated exercise (panel D).

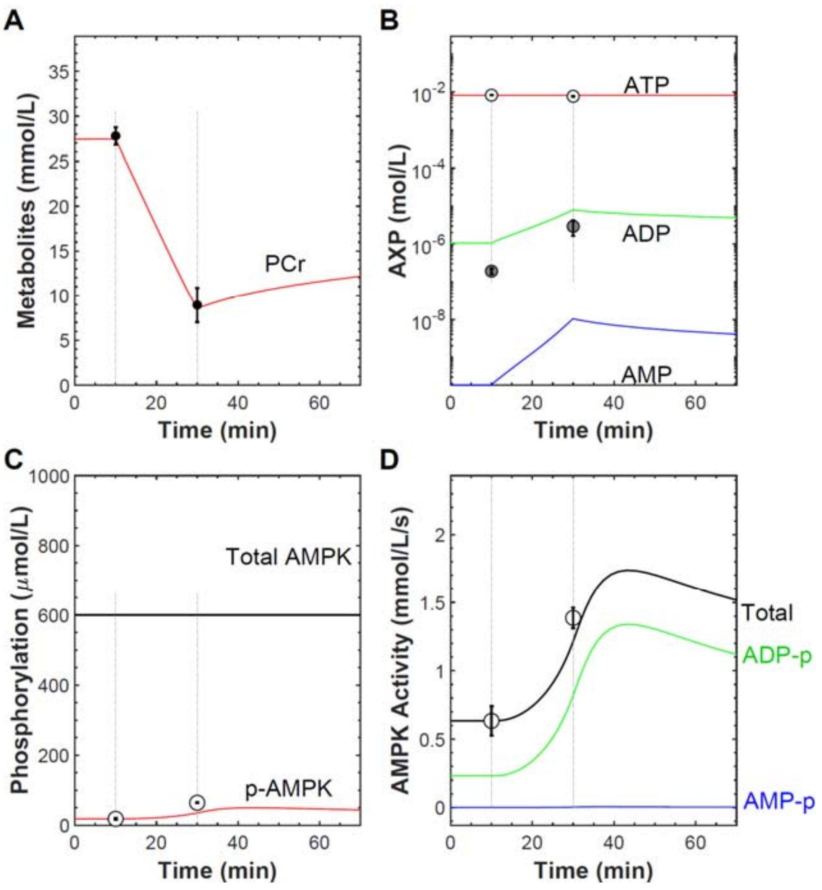

Figure 2. Simulation of the Nielsen Sedentary protocol with lower ADP levels (resting = ~1 μM, i.e., 10^{-6} M; panel B). In this simulation, the K_{eqCK} was increased to 3,925 from its nominal value of 111. k_{stim} and k_{rest} were then adjusted to ensure the fit of the PCr data. ADP-p-AMPK levels (green line) were increased relative to those of AMP-p-AMPK (blue line) during simulated exercise (panel D).

References

1. Ross FA, Jensen TE, Hardie DG. Differential regulation by AMP and ADP of AMPK complexes containing different γ subunit isoforms. Biochem J. 2016;473: 189–99. doi:10.1042/BJ20150910

2. Suter M, Riek U, Tuerk R, Schlattner U, Wallimann T, Neumann D. Dissecting the role of 5'-AMP for allosteric stimulation, activation, and deactivation of AMP-activated protein kinase. J Biol Chem. 2006;281: 32207–16. doi:10.1074/jbc.M606357200

3. Gowans GJ, Hawley SA, Ross FA, Hardie DG. AMP is a true physiological regulator of AMP-activated protein kinase by both allosteric activation and enhancing net phosphorylation. Cell Metab. 2013;18: 556–66. doi:10.1016/j.cmet.2013.08.019

4. Langendorf CG, Ngoei KRW, Scott JW, Ling NXY, Issa SMA, Gorman MA, et al. Structural basis of allosteric and synergistic activation of AMPK by furan-2-phosphonic derivative C2 binding. Nat Commun. Nature Publishing Group; 2016;7: 1–8.
